# Synthesis and characterization of thymol-derived phenoxy acetamide derivatives using DFT, molecular docking, and parasitological investigations

**DOI:** 10.3389/fchem.2025.1579923

**Published:** 2025-05-21

**Authors:** Ahmed R. Rabee, Saied M. Soliman, Hamida Abdel-Hamid, Esraa A. Moneer, Sara H. Akl, Yahya H. Shahin, Aliaa A. Masoud, Doaa Ahmad Ghareeb, Mariusz Jaremko, Abdul-Hamid Emwas, Mazen Sherif, Mohamed Hagar

**Affiliations:** ^1^ Chemistry Department, Faculty of Science, Alexandria University, Alexandria, Egypt; ^2^ Department of Medical Laboratory Technology, Faculty of Applied Health Sciences Technology, Pharos University in Alexandria, Alexandria, Egypt; ^3^ Department of Medical Laboratory Technology, Faculty of Health and Medical Techniques, Almaaqal University, Basrah, Iraq; ^4^ Bio-Screening and Preclinical Trial Lab, Biochemistry Department, Faculty of Science, Alexandria University, Alexandria, Egypt; ^5^ Biological and Environmental Sciences and Engineering Division, King Abdullah University of Science and Technology, Makkah, Saudi Arabia; ^6^ KAUST Core Laboratories, King Abdullah University of Science and Technology, Thuwal, Saudi Arabia

**Keywords:** thymol, naphthalimide and phthalimide derivatives, anti-parasites, molecular docking, DFT

## Abstract

Novel phenoxy acetamide derivatives based on a thymol moiety were synthesized for target parasitological investigation. The newly synthesized compounds, **5a**, **5b**, **7a**, **7b**, and **9**, were synthesized as phenoxy acetamide derivatives containing a phthalimide or naphthalimide ring through a condensation reaction with various acid anhydrides. Their structures were confirmed based on spectral data derived through Fourier-transform infrared, proton and carbon-13 nuclear magnetic resonance, and elemental analyses. The parasitological, biochemical, and immunological activities of the compounds were measured. The screened compounds were subjected to molecular docking in the active site of CpCDPK1, in addition to analyses based on Lipinski’s rule and SwissADME. The results showed that compounds **5a**, **5b**, and **7b** demonstrated promising antiparasitic activity, characterized by high gastrointestinal absorption and favorable drug-likeness profiles. Furthermore, **5a** and **7b** exhibited higher binding affinities than that of the reference drug. In practical assessments, compound **7b** exhibited the highest percentage reduction in oocyst counts (67%). Density functional theory calculations were performed to assess the thermodynamic stability, molecular geometry, frontier molecular orbital energy gaps, and molecular electrostatic potentials of compounds **5a**, **5b**, **7a**, **7b**, and **9**.

## 1 Introduction

Thymol (2-isopropyl-5-methylphenol) is a white crystalline aromatic compound with a distinct scent that is produced *via* the terpene pathway and typically found in the genera *Thymus* and *Origanum*. Its direct biosynthetical link with p-cymene has been demonstrated through the aromatization of c-terpinene in *Thymus vulgaris* L. (Poulose and Croteau, 1978) According to the United States Food and Drug Administration (US-FDA), thymol is safe with low toxicity. (Cohen, 1984). Thymol has been known to exert therapeutic effects on humans, (Jyoti et al., 2019), with a range of biological activities, such as antibacterial, (Dong et al., 2024), antifungal, (Alves Eloy et al., 2021), antioxidant, (Sathe et al., 2019), antiprotozoal, (Clemente et al., 2021), anti-inflammatory, (Chen et al., 2020), antiviral, (Nandi and Khanna, 2022), antiparasitic, (Hikal et al., 2021; Sennouni et al., 2022), and anticancer (Lv and Chen, 2017; Chauhan et al., 2017) activities. Additionally, thymol has shown efficacy against *C. parvum (Cryptosporidium parvum)* in cell cultures. (Dominguez-Uscanga et al., 2021).

Phenoxy acetamide derivatives exhibit antioxidant activity and may protect biological systems against oxidative damage. (Al-Ostoot et al., 2021a; Arshad et al., 2022). Molecules containing acetamide linkages and their derivatives as core structures display a wide range of biological activities, (Al-Ostoot et al., 2021b), especially antitrypanosomal effects. (Capelini et al., 2021). Phthalimides, which are neutral, lipophilic compounds exhibiting antimicrobial (Akgün et al., 2012) and anti-inflammatory (Alanazi et al., 2015) properties readily penetrate biological membranes. Thus, phthalimides have been used as platforms for developing anti-*Trypanosoma cruzi* and antiplasmodial drugs. (González et al., 2014; Singh et al., 2015).

Naphthalimide derivatives have conventionally been used as fluorescent fibers and dyes. In prior decades, these derivatives were used as medicines and sensors. (Qian et al., 2010). The tricyclic planar ring structure of the naphthalimide class facilitates intercalation with DNA and disruption of biological processes. (Kamal et al., 2013). Hence, naphthalimides exhibit a range of biological attributes, such as antiviral, (Chanh et al., 1994), antioxidant, (Ghosh et al., 2015), and antimicrobial activities. (Staneva et al., 2019).

Thymol, characterized as a safe compound, (Aljohani et al., 2022), exhibits various advantages, such as abundance and low toxicity to mammalian cells. Consequently, thymol and related substances may represent promising candidates for developing novel antiparasitic therapies (Chauhan and Kang, 2014).

The zoonotic protozoan parasite *C. parvum*, which is the second-most common cause of foodborne and waterborne diarrheal illnesses globally, is a member of the phylum Apicomplexa. (Ryan et al., 2016). Its adverse effects are particularly severe for immunocompromised individuals. (Wang et al., 2018).

Considering these aspects, this study was aimed at synthesizing newly phenoxy acetamide derivatives based on a thymol moiety through condensation reactions with a series of acid anhydrides and assessing their antiparasitic effects through experimental and molecular docking studies. One of the challenges with thymol is its rapid absorption and metabolism, which can limit its effectiveness *in vivo*. The phenoxy acetamide derivatives based on thymol can potentially create prodrugs that release thymol more slowly, allowing for sustained therapeutic effects. Moreover, density functional theory (DFT) calculations were performed to explore their thermodynamic stability, molecular geometry, frontier molecular orbital (FMO) energy gaps, and molecular electrostatic potential (MEP).

## 2 Results and discussion

### 2.1 Chemistry

**SCHEME 1:**
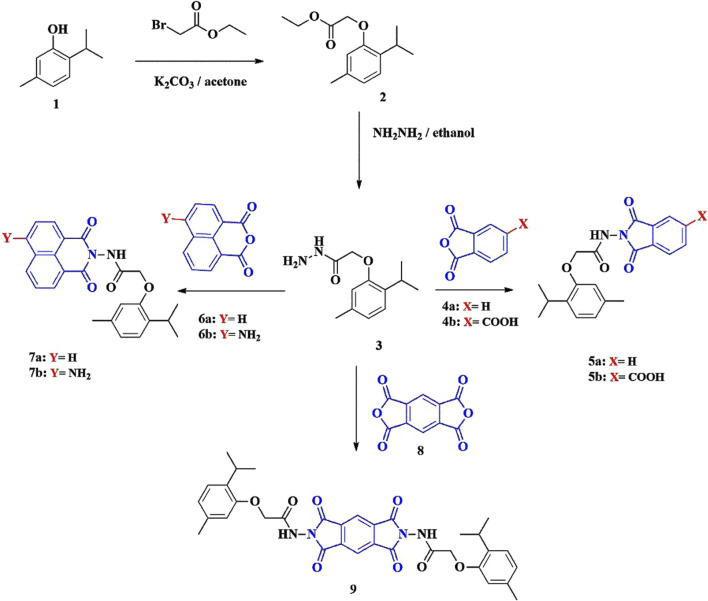
Synthesis of phenoxy acetamide derivatives containing a thymol unit.

Phenoxy acetamide derivatives based on a thymol moiety were synthesized through condensation reactions with a series of acid anhydrides. As shown in Scheme 1, 2-(2-isopropyl-5-methylphenoxy)acetohydrazide (**3**) was reacted with different acid anhydrides, including phthalic anhydride (**4a**), 1,2,4-benzene tricarboxylic acid anhydride (**4b**), 1,8-naphthalic anhydride (**6a**), 4-amino-1,8-naphthalic anhydride (**6b**), and pyromellitic dianhydride (**8**) in dimethylformamide (DMF) and glacial acetic acid under reflux conditions for 4–6 h to afford compounds **5a**, **5b**, **7a**, **7b**, and **9**, respectively.

The structures of the synthesized compounds **5a**, **5b**, **7a**, **7b**, and **9** were confirmed using spectroscopic data. The proton nuclear magnetic resonance (^1^H-NMR) spectrum showed characteristic singlet peaks for NH (10.08–10.90 ppm) and aliphatic CH_3_ (1.13–2.27 ppm). The infrared (IR) spectra of all synthesized compounds showed two sharp bands of C=O (conjugated anhydride) at *ν* = 1,792 cm^−1^ and 1,744 cm^−1^.

For compound **5b**, the ^1^H-NMR spectrum showed a singlet peak of COOH at 10.13 ppm and the ^13^C-NMR spectrum showed a peak at *δ*_C_: 168.4 (COOH). The ^1^H-NMR spectrum of compound **7b** showed a singlet peak of NH_2_ at 7.71 ppm. For compound **9**, the ^1^H-NMR spectrum showed two singlet peaks at 8.45 and 8.29 ppm, corresponding to pyromellitic protons.

### 2.2 *In silico* physicochemical and pharmacokinetic predictions

An absorption, distribution, metabolism, excretion, toxicity (ADMET) study was performed to assess the pharmacokinetic, physicochemical, and drug-likeness profiles of compounds **5a**, **5b**, **7a**, **7b**, and **9**. As indicated in Table 1, compounds **5a**, **5b**, **7a**, and **7b** demonstrated high gastrointestinal (GI) absorption, whereas compound **9** exhibited low GI absorption. Blood–brain barrier (BBB) permeability was observed only for **5a** and **7a**. None of the compounds were substrates for P-glycoprotein. Selective inhibition of cytochrome P450 enzymes was noted, with compound **5b** inhibiting CYP1A2 and compounds **5a**, **7a**, and **7b** inhibiting CYP2C19. Furthermore, all compounds inhibited CYP2C9 and compound **9** uniquely inhibited CYP3A4. Drug-likeness analysis revealed no violations of Lipinski’s rule of five for **5a**, **5b**, **7a**, and **7b**, although compound **9** exhibited two violations. Bioavailability scores were consistent for **5a**, **5b**, **7a**, and **7b**, ranging from 0.55 to 0.56, whereas compound 9 had a significantly lower score (0.17). Medicinal chemistry assessments indicated that **5b** had the fewest lead-likeness violations, followed by **5a**, **7a**, and **7b**, with compound **9** exhibiting the most violations. Synthetic accessibility scores showed that **5a** was the easiest to synthesize and **9** posed the greatest challenge. Physicochemical analysis revealed that compound **9** had the highest molecular weight (626.66 g/mol) and topological polar surface area (TPSA) (154.8 Å^2^), correlating with low membrane permeability. Compound **5a** exhibited the lowest molecular weight and TPSA, contributing to its favorable pharmacokinetic profile. Compound **9** also had the highest number of rotatable bonds, indicating greater molecular flexibility, while **5a**, **7a**, **7b** was more rigid. Lipophilicity was most pronounced in compound **9** (consensus log Po/w of 4.06) and **5b** exhibited the lowest value (2.54). Solubility predictions categorized **5a**, **5b**, **7a**, and **7b** as moderately soluble, while **9** was noted to be poorly soluble (ESOL log S of −6.64). Among the tested compounds, **5a, 7a, 7b** emerged as the most promising owing to their high GI absorption, moderate solubility (ESOL log S of −4.24, −5.46, −5.11), balanced lipophilicity (consensus log Po/w 2.95, 3.91, 3.39), and TPSA (75.71, 77.4, 103.42 Å^2^). It selectively inhibited CYP2C19 and CYP2C9 and did not interact with CYP1A2, CYP2D6, or P-gp, resulting in enhanced therapeutic safety profile. However, compound **7b** stands out as a safer candidate due to its lack of BBB permeability, which may reduce potential central nervous system side effects.

**TABLE 1:** ADMET study of compounds **5a**, **5b**, **7a**, **7b**, and **9.**

Pharmacokinetics	5a	5b	7a	7b	9
GI absorption	High	High	High	High	Low
BBB permeability	Yes	No	Yes	No	No
P-gp substrate	No	No	No	No	No
CYP1A2 inhibitor	No	Yes	No	No	No
CYP2C19 inhibitor	Yes	No	Yes	Yes	No
CYP2C9 inhibitor	Yes	Yes	Yes	Yes	Yes
CYP2D6 inhibitor	No	No	No	No	No
CYP3A4 inhibitor	No	No	No	No	Yes

Drug Likeness	5a	5b	7a	7b	9
Lipinski violations	0	0	0	0	2
Bioavailability score	0.55	0.56	0.55	0.55	0.17

Medicinal Chemistry	5a	5b	7a	7b	9
Lead likeness violations	2	1	2	2	3
Synthetic accessibility	2.75	2.98	3.08	3.19	4.27

Physicochemical Properties	5a	5b	7a	7b	9
Formula	C_20_H_20_N_2_O_4_	C_21_H_20_N_2_O_6_	C_24_H_22_N_2_O_4_	C_24_H_23_N_3_O_4_	C_34_H_34_N_4_O_8_
MW	352.38	396.39	402.44	417.46	626.66
Num. heavy atoms	26	29	30	31	46
Num. aromatic heavy atoms	12	12	19	19	24
Fraction Csp3	0.25	0.24	0.21	0.21	0.29
Num. rotatable bonds	6	7	6	6	12
Num. H-bond acceptors	4	6	4	4	8
Num. H-bond donors	1	2	1	2	2
MR	100.09	107.05	118.78	123.18	176.11
TPSA	75.71	113.01	77.4	103.42	154.8

Lipophilicity	5a	5b	7a	7b	9
Consensus log *P*_o/w_	2.95	2.54	3.91	3.39	4.06

Water Solubility	5a	5b	7a	7b	9
ESOL log S	−4.24	−4.11	−5.46	−5.11	−6.64
ESOL solubility (mg/mL)	2.04E^-02^	3.10E^-02^	1.38E^-03^	3.22E^-03^	1.44E^-04^
ESOL solubility (mol/L)	5.78E^-05^	7.81E^-05^	3.44E^-06^	7.71E^-06^	2.29E^-07^
ESOL class	Moderately soluble	Moderately soluble	Moderately soluble	Moderately soluble	Poorly soluble

### 2.3 Molecular docking studies

**FIGURE 1:**
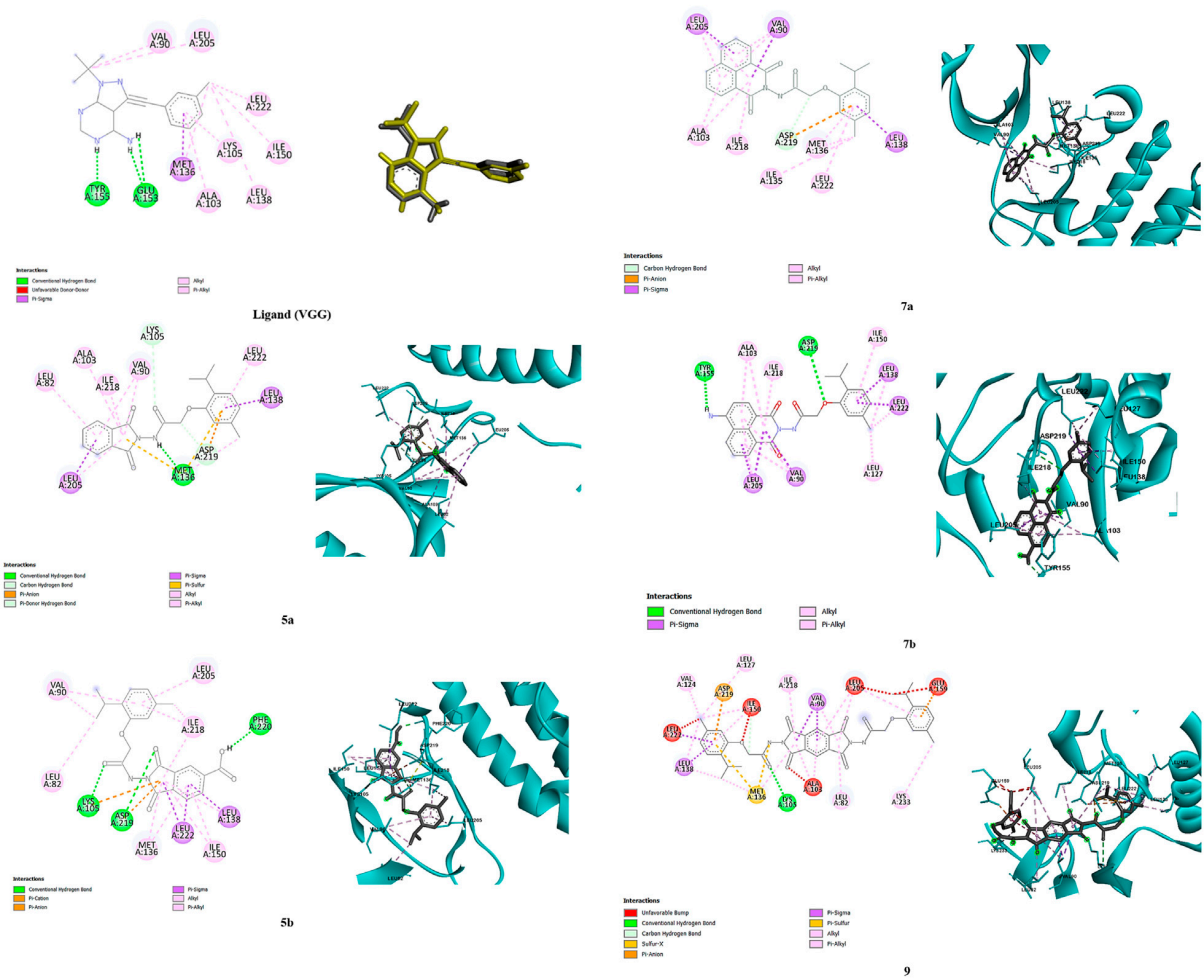
Binding poses of compounds **5a**, **5b**, **7a**, **7b**, and **9**, and ligand (VGG) in the 2WEI active site (2D and 3D representations).

Molecular docking results were validated by re-docking the native ligand VGG into the CDPK1 active site, considered a key target for antiparasitic drug development owing to its essential role in the lifecycle of parasites. The analysis yielded a root mean square deviation (RMSD) of 0.52 Å and docking score of −7.27 kcal/mol, validating the proposed methodology. The binding affinity of each compound (**5a**, **5b**, **7a**, **7b**, and **9**) is outlined in Table 2. As shown in Figure 1, compound **5a** demonstrated the highest binding affinity (−7.42 kcal/mol), forming strong hydrogen bonds with critical catalytic residues Asp219, Met136 and Lys105, along with stabilizing π-alkyl and van der Waals interactions highlighting its potential as a potent inhibitor. Compound **5b** showed the second-highest binding affinity (−6.45 kcal/mol) and interacted with Asp219, Lys105, and Phe220, indicating a slightly weaker but still significant binding conformation. Compound **7b** exhibited a comparable binding affinity (−6.41 kcal/mol), with conventional hydrogen bond interactions involving Tyr155 and Asp219, suggesting a stable binding profile. In contrast, compounds **7a** and **9** displayed notably weaker binding profiles. Compound **7a** had a lower binding affinity (−5.39 kcal/mol), with limited hydrogen bond interactions involving Asp219, reflecting a less stable binding mode. Compound **9** demonstrated a highly unfavorable binding affinity (32.66 kcal/mol) owing to steric clashes and unfavorable conformations within the active site, indicating a lack of inhibitory potential. Although compound **7b** had a slightly lower binding affinity than **5a** and **5b**, it demonstrated the highest *in vivo* antiparasitic activity, attributable to its balanced ADMET profile, including high GI absorption, moderate solubility, and selective cytochrome P450 inhibition, which enhanced its bioavailability and efficacy. In our *in silico* analyses, compounds **5a, 7a,** and **7b** emerged as the most promising candidates; however, compound **7b** was particularly notable because, in addition to its favorable ADMET properties, it selectively bound to Tyr155 in the active site which is the same residue to which the co-crystallized ligand VGG binds. This selective interaction suggests that Tyr155 plays a key role in CpCDPK1 activity, and even though **7b** exhibited slightly lower binding energy, its engagement with Tyr155 likely contributes to its enhanced antiparasitic effects. Conversely, the experimental results of compounds 7a and 9, which showed the lowest antiparasitic activities, correlated with their poorer docking scores and weaker binding interactions within the active site. These findings underscore the critical role of stable binding interactions, particularly with key residues, and favorable pharmacokinetic profiles in achieving potent *in vivo* activity, thereby highlighting compound 7b as a promising candidate for further drug development.

**TABLE 2:** Docking scores of compounds and types of interactions with 2WEI.

Ligand compound	Binding energy score (S) (Kcal/mol)	RMSD (RefRMSD)	Inhibition constant (Ki) ( $\left.\mu M\right)$	Type of binding interactions	Residues involved in the interactions
Ligand (VGG)	−7.27	0.52	4.7	Hydrogen bonds	Pyrimidine nitrogen with Tyr155, amine hydrogen with Glu153.
5a	−7.42	0.0	3.65	Hydrogen bond	Methyl hydrogen with Asp219, carbonyl oxygen with Lys105, and amide proton with Met136.
5b	−6.45	0.0	18.6	Hydrogen bond	Carbonyl oxygen with Lys105, carboxyl proton with Phe220, and carbonyl oxygen with Asp219.
7a	−5.39	0.0	111.9	Hydrogen bond	Methyl hydrogen with Asp219.
7b	−6.41	0.0	20.03	Hydrogen bond	Amine with Tyr155 and ether oxygen with Asp219
9	32.66	0.0	0.0	Unfavorable bumps	Multiple bumps

### 2.4 Biological evaluation

#### 2.4.1 Cytotoxic effects on red blood cells (RBCs)

The *in vitro* cytotoxic effects of the newly synthesized compounds on RBCs were assessed through hemolytic activity. The hemolysis percentages at 1,000 μg/mL were zero for **5a**, **5b**, and **7b**; 6.18% for **7a**; and 34.95% for **9** (Table 3)**.**

**TABLE 3:** *In vitro* hemolytic activity and IC_50_ values (cytotoxicity on RBCs).

Concentration (μg/mL)	Hemolysis %				
	5a	7a	5b	7b	9
62.5	0	0	0	0	0
125	0	0	0	0	0
250	0	0	0	0	0
500	0	5.0167	0	0	0
1,000	0	6.1873	0	0	34.95
IC _50_ (μg/mL)	ND	6754.9	ND	ND	1,556.6

IC_50_ (μg/mL): sample concentration causing 50% hemolysis of RBCs. ND, not determined.

#### 2.4.2 Parasitological results

**FIGURE 2:**
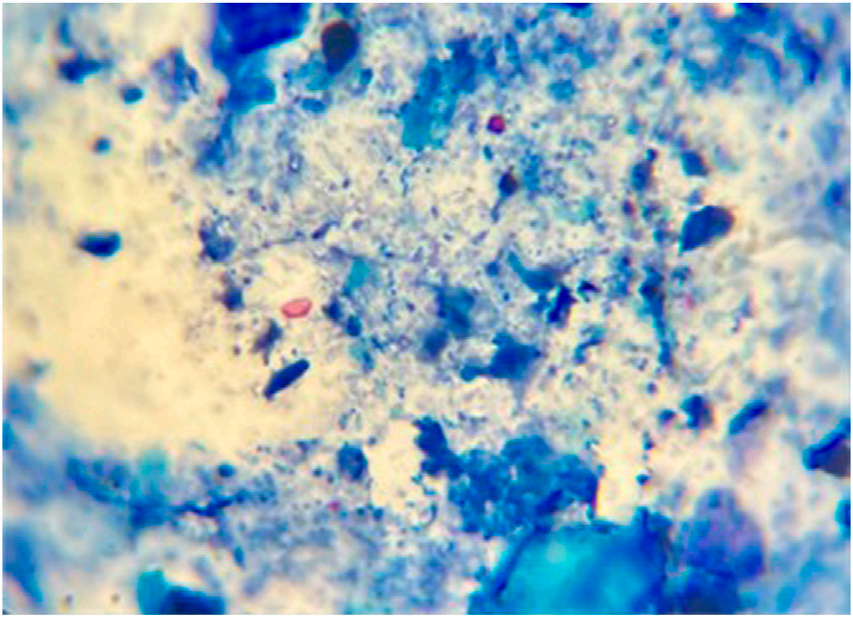
Fecal smear stained with modified Ziehl–Neelsen stain, showing *Cryptosporidium* oocysts (black arrows). Magnification: 1,000×.

Using the modified Ziehl–Neelsen staining technique, oocysts were identified as bright-pink oval or round structures against a bluish background (Figure 2).

Table 4 indicates significant statistical differences among all groups. The mean oocyst count in the infected, non-treated group (G2) was 34.4 ± 0.69, while the corresponding value in the nitazoxanide (NTZ)-treated group was 10.4 ± 0.51, indicating the highest reduction in oocyst count. Among the tested compounds, the **7b**- and **7a**-treated groups exhibited the highest (67%) and lowest (51%) percentage reduction in the oocyst count, respectively.

**TABLE 4:** Number of *Cryptosporidium*-oocyst-shedding immunosuppressed mice in different study groups.

Shed oocysts/mg	G2	G3	G 4	G5	G6	G7	G8	F	P
Mean ± SD	34.4 ± 0.69	10.4 ± 0.51	13.2 ± 1.034	14.7 ± 0.67	16.8 ± 0.63	11.5 ± 0.85	15.4 ± 0.52	389.597	0.000
(R%)		70%	62%	57%	51%	67%	55%		
P within groups		P_1_ 0.000**	P_2_ 0.000**	P_3_ 0.000**	P_4_ 0.000**	P_5_ 0.000**	P_6_ 0.000**		
			P_7_ 0.004*	P_8_ 0.000**	P_9_ 0.000**	P_10_ 0.519^ns^	P_11_ 0.000**		
				P_12_ 0.022*	P_13_ 0.000**	P_14_ 0.011*	P_15_ 0.002*		
					P_16_ 0.003*	P_17_ 0.000**	P_18_ 0.250^ns^		
						P_19_ 0.000**	P_20_ 0.031*		
							P_21_ 0.000**		

F: One-way ANOVA, test.

P_1_: G2 vs G3, P_2_: G2 vs G4, P_3_: G2 vs G5, P_4_: G2 vs G6, P_5_: G2 vs G7, P_6_: G2 vs G8, P_7_: G3 vs G4, P_8_: G3 vs G5, P_9_: G3 vs G6, P_10_: G3 vs G7, P_11_: G3 vs G8, P_12_: G4 vs G5, P_13_: G4 vs G6, P_14_: G4 vs G7, P_15_: G4 vs G8, P_16_: G5 vs G6, P_17_: G5 vs G7, P_18_: G5 vs G8, P_19_: G6 vs G7, P_20_: G6 vs G8 and P_21_: G7 vs G8.

R%: reduction percent in oocyst count.

Ns not significant, * significant at p < 0.05, ** significant at p < 0.001.

##### 2.4.2.1 Ultrastructural analysis

**FIGURE 3:**
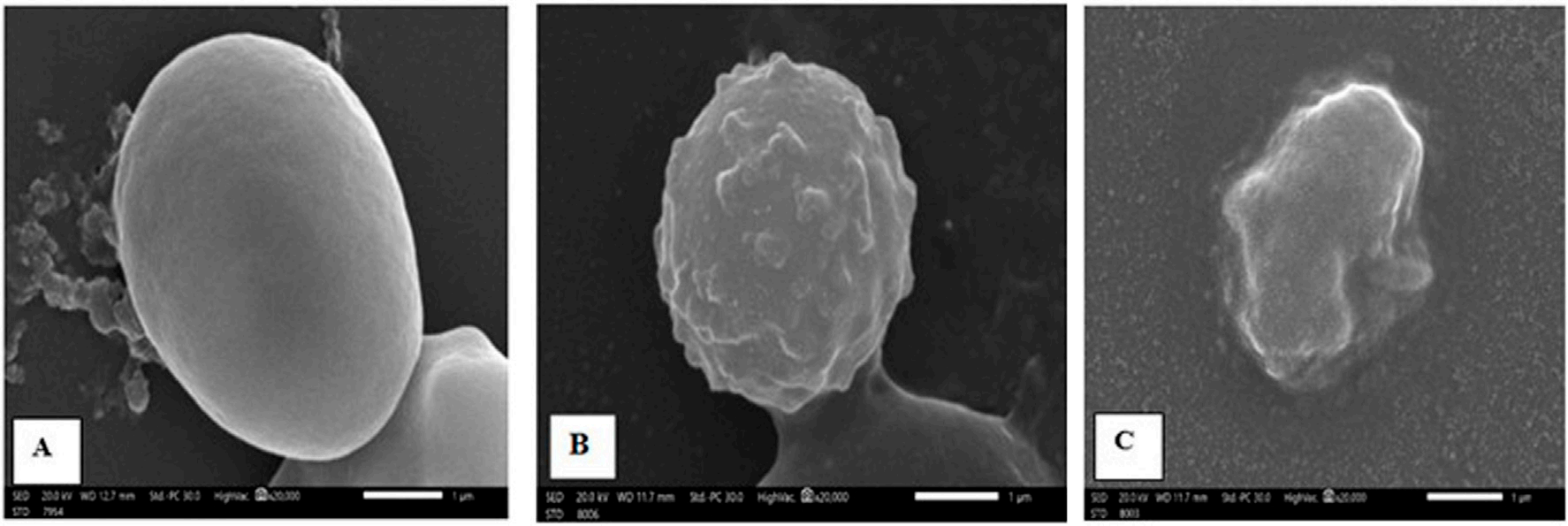
Scanning electron micrography images of *Cryptosporidium* oocysts from the **(A)** infected control group (G2), displaying a spherical shape with an extremely smooth surface; **(B)** NTZ-treated group (G3), showing rough, irregular surfaces with bleb formation; and **(C) 7b**-treated group, exhibiting complete distortion with irregular shapes and outlines.

Scanning electron microscopy (SEM) was used to evaluate the ultrastructural morphological features of oocysts in fecal samples for each group. Oocysts from the control group exhibited a uniform spherical shape with smooth cyst surfaces. In contrast, cysts extracted from treated mice exhibited varying degrees of morphological changes (Figure 3).

##### 2.4.2.2 Transmission electron microscopy (TEM)

**FIGURE 4:**
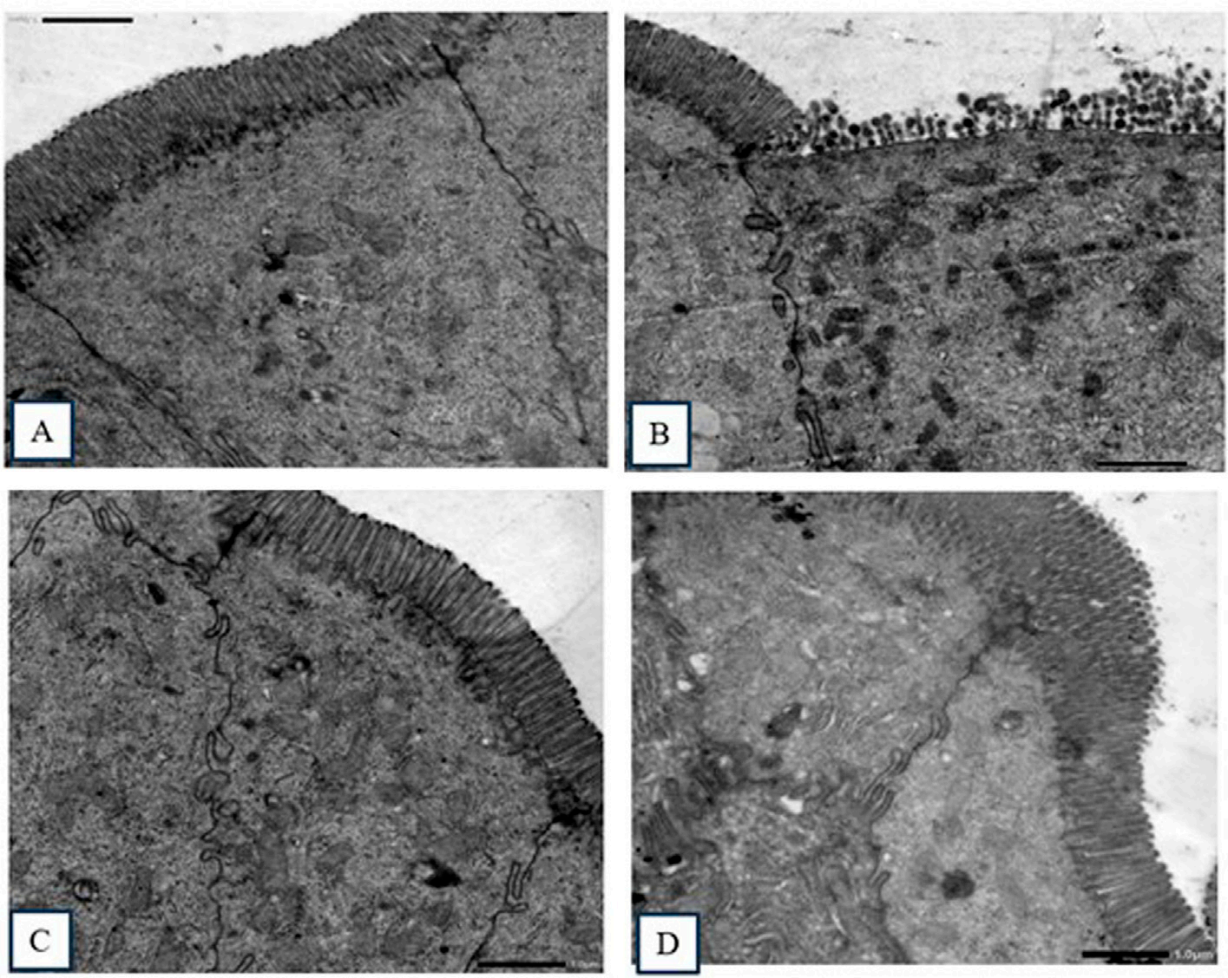
Transmission electron micrography images of sections in the small intestine. **(A)** Non-infected, non-treated group, showing intestinal columnar epithelial cells with normal prominent microvilli and well-developed cell junctions. **(B)** Infected, non-treated group, showing ileal epithelial cells with significantly damaged epithelial lining and distorted and blunted microvilli. **(C)** NTZ-treated group, showing normal ultrastructure and regularly arranged apical microvilli with oval euchromatic nuclei. **(D) 7b**-treated group, showing healthy microvilli (slightly disfigured and disarranged).

TEM images (Figure 4) of ultrathin sections of small intestinal epithelial cells from the non-infected, non-treated control group revealed typical prominent microvilli, regular euchromatic nuclei, and well-developed cell junctions. In contrast, all treated groups revealed different levels of abnormalities, ranging from slight disarrangement to completely damaged atrophied microvilli.

#### 2.4.3 Biochemical measurements

Biochemical parameter levels were significantly increased in the infected, non-treated control group compared with the negative control group. All treated groups showed significant changes in the biochemical parameter levels compared with the positive control group (Table 4).

#### 2.4.4 Immunological measurements

In the serum cytokine analysis (Table 5), the levels of interferon-gamma (IFN-γ), tumor necrosis factor-alpha (TNF-α), interleukin-6 (IL-6), and interleukin-10 (IL-10) were significantly higher (*P* < 0.001) in the infected control group compared with the negative control group. The mean levels of IFN-γ, IL-6, and IL-10 were significantly reduced (*P* < 0.001) in the treated groups compared with the infected control group (Table 6).

**TABLE 5:** Cytokine levels in mice groups.

Group	TNF-α (pg/mL)	IFN-γ (pg/mL)	IL-6 (pg/mL)	IL-10 (pg/mL)
G1	9.3 ± 0.94	342.5 ± 4.16	88.1 ± 0.87	253.4 ± 1.17
G2	33.25 ± 0.79	717.4 ± 2.67	260.5 ± 0.70	397.4 ± 1.42
G3	7.8 ± 0.91	422.1 ± 3.21	111.4 ± 1.17	276.5 ± 0.97
G4	13.1 ± 0.99	477.8 ± 1.61	176.8 ± 0.91	327.6 ± 1.34
G5	13.7 ± 1.25	494.2 ± 3.15	181.7 ± 149	387.8 ± 0.78
G6	15.9 ± 0.87	608.8 ± 2.48	237.4 ± 2.23	434.2 ± 1.13
G7	11.8 ± 1.03	415.2 ± 2.57	143.1 ± 1.1	277.2 ± 1.47
G8	16.2 ± 1.03	534.2 ± 1.26	215.7 ± 1.15	416.8 ± 0.63
F	165.828*	1,232.828*	736.518*	1,224.031*
P	<0.001*	<0.001*	<0.001*	<0.001*

F, One-way ANOVA *P value < 0.05 significant.

**TABLE 6:** Biochemical analysis of mice groups.

Group	ALT (U/L)	AST (U/L)	Urea (mg/dL)	Creatinine (mg/dL)	Total protein (g/dL)	Albumin (g/dL)
G1	8.11 ± 0.61	25 ± 0.56	19 ± 0.89	0.15 ± 0.93	2.32 ± 1.21	4.1 ± 1.32
G2	14.35 ± 0.63	68.32 ± 0.85	41.42 ± 0.98	0.69 ± 0.39	2.62 ± 0.96	5.16 ± 1.11
G3	25.69 ± 0.92	86.36 ± 0.81	22.59 ± 0.93	1.26 ± 0.73	2.33 ± 0.68	4.56 ± 0.71
G4	24.6 ± 1.22	97.55 ± 0.55	26.45 ± 0.51	1.36 ± 0.14	2.46 ± 0.43	4.63 ± 0.93
G5	23.58 ± 0.08	94.63 ± 0.26	24.33 ± 0.38	1.52 ± 0.45	2.49 ± 1.22	4.87 ± 1.12
G6	39.68 ± 0.82	130.5 ± 0.67	45.34 ± 0.36	1.76 ± 0.36	2.4 ± 0.62	4.8 ± 0.84
G7	21.53 ± 0.31	95.63 ± 1.63	25.3 ± 0.92	0.96 ± 0.71	2.53 ± 0.64	4.74 ± 0.38
G8	30.21 ± 0.64	105.96 ± 0.81	35.85 ± 0.63	1.46 ± 0.33	2.32 ± 0.47	4.66 ± 0.41
F	122.815	667.930	597.905	136.462	321.651	14.631
P	<0.001*	<0.001*	<0.001*	<0.001*	<0.001*	<0.001*

F, One-way ANOVA, *P value < 0.05 significant.

### 2.5 DFT study

#### 2.5.1 Geometrical structure

Compounds **5a**, **5b**, **7a**, **7b**, and **9** were theoretically examined using DFT calculations. Gauss View 6.016 (Dennington et al., 2016) was used to draw the molecular structures. Computations were performed in the gas phase using Gaussian 09 Revision D.01 software, (Caricato et al., 2009), adopting the DFT/B3LYP method at a 6–311 g basis set.

**FIGURE 5:**
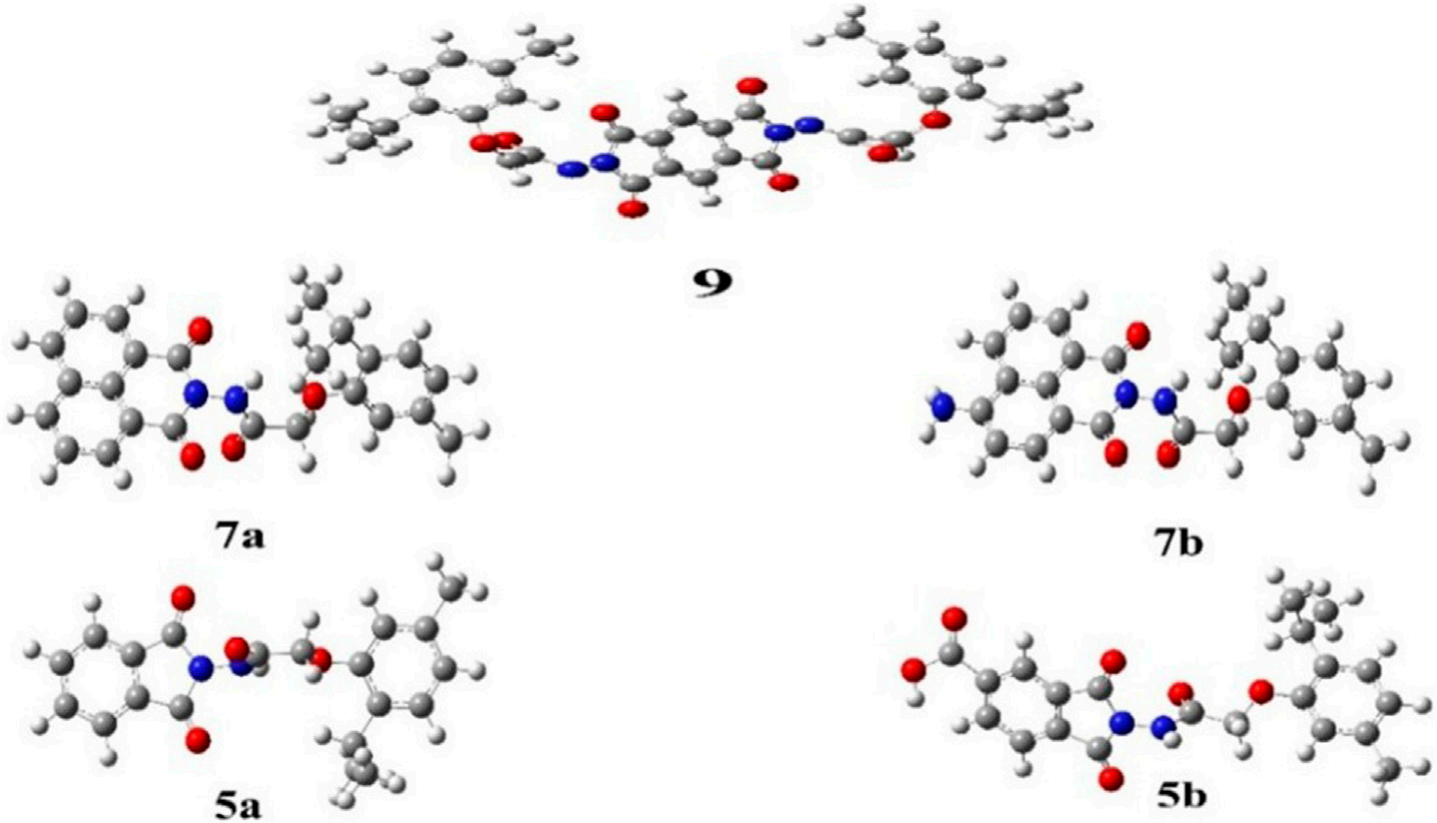
Optimized geometrical structures of synthesized phenoxy acetamide derivative compounds **5a**, **5b**, **7a**, **7b**, and **9** (gray, carbon; white, hydrogen; red, oxygen, and blue, nitrogen).

This method facilitated the optimization of the molecular geometry to determine the lowest energy and most stable structures, commonly referred to as convergence. In addition, a frequency process was applied to the optimized structures using the same basis sets to compute their thermodynamic parameters. The absence of imaginary frequencies confirmed the stability of the optimized compounds, and all synthesized compounds displayed non-coplanar structures. The optimized compounds are presented in Figure 5.

#### 2.5.2 Polarizability and dipole moment

Molecular polarizability refers to the degree to which the electron cloud of a molecule can be distorted by an external charge, leading to the development of an electric dipole moment. (Katariya et al., 2024). The electronic nature and polarity of terminal substituents considerably affect the polarizability and dipole moment of the compound. (Soni et al., 2024). The calculated polarizability values for the investigated compounds are presented in Table 7.

**TABLE 7:** Calculated polarizability (a.u.) and dipole moment **(Debye)** of compounds **5a**, **5b**, **7a**, **7b**, and **9.**

Compound	Dipole moment				Polarizability
	X	Y	Z	Total	
5a	−0.4286	−0.7157	−1.9086	2.0830	244.40
5b	−0.6944	−6.1862	0.1809	6.2277	265.59
7a	−3.0859	1.4869	1.3867	3.6955	295.54
7b	5.5612	0.8400	−0.9210	5.6992	310.61
9	−1.9875	3.3012	1.8926	4.2930	484.64

Dipole moment calculations were conducted along the x, y, and z axes. It is observed that **5a** which contains only one phthalimide ring has a very low dipole moment. Meanwhile, **5b** and **7b**. have higher dipole moment values due to the presence of COOH and NH_2_ groups.

#### 2.5.3 MEP mapping

MEP mapping serves as a valuable tool for assessing electron density and charge distribution in a molecule, enabling the prediction of intermolecular and intramolecular interactions, as well as the extent of molecular packing and formal and partial charge of atoms. (Butera, 2024). Following the same basis set, charge distribution maps for all compounds were derived. In the maps, regions of electron density were illustrated in ascending order: red > orange > yellow > green > blue. Specifically, regions with high electronegativity appeared in red, whereas regions with the least negative charge appeared in blue. (Politzer and Murray, 2021).

**FIGURE 6:**
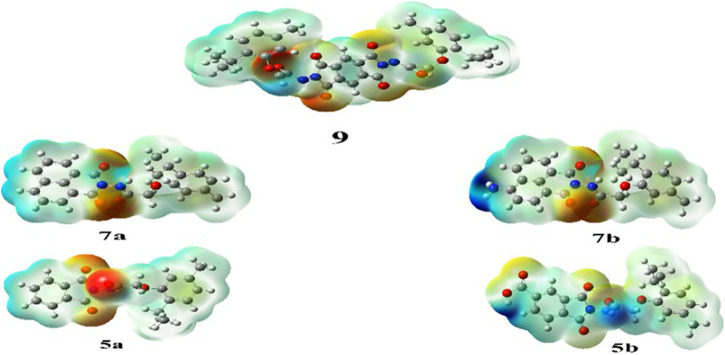
Molecular electrostatic potential (MEP) maps of synthesized compounds.

All synthesized compounds (**5a**, **5b**, **7a**, **7b**, and **9**) exhibited a high negative charge in the center regions owing to the presence of acetamide and phthalimide groups, each contributing a half-negative charge on their oxygen atoms. In compound **5b**, the highest negative charge was localized on the oxygen atom of the COOH group. The MEP maps are shown in Figure 6.

#### 2.5.4 FMO analysis

**FIGURE 7:**
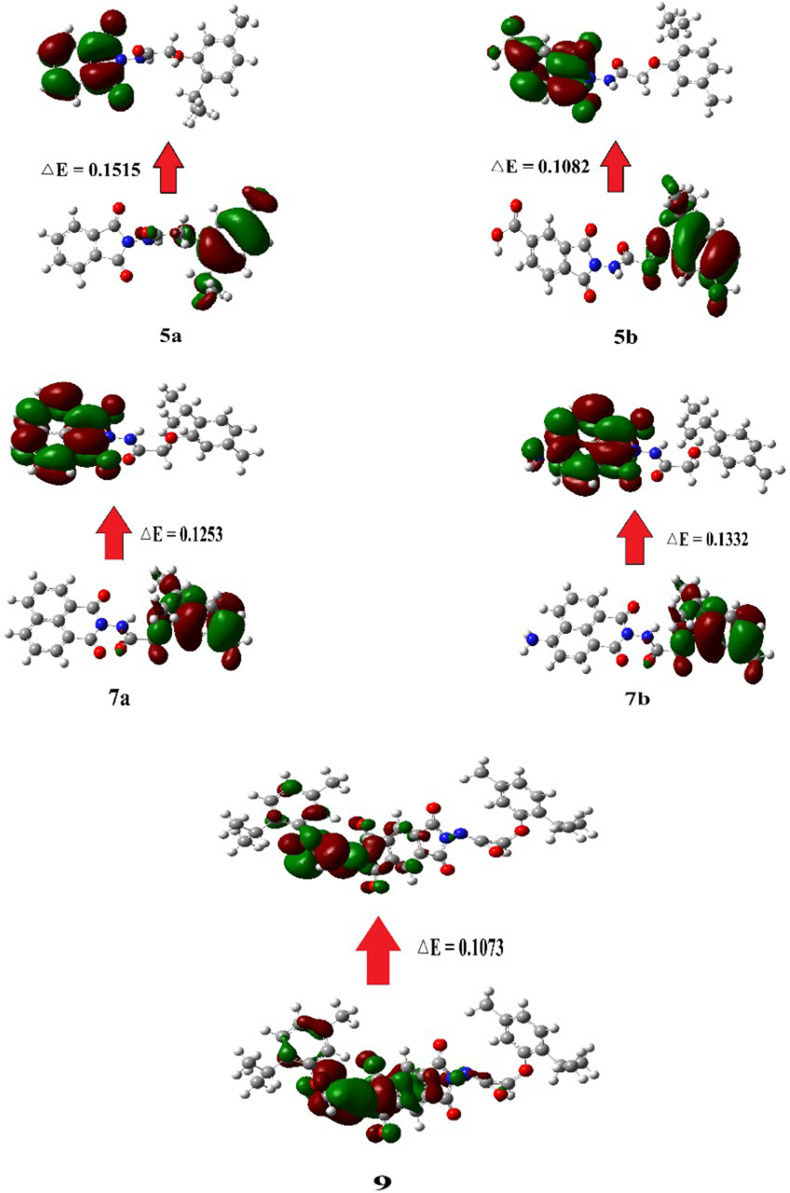
Graphical representation of the frontier molecular orbitals (FMOs) of compounds **5a**, **5b**, **7a**, **7b**, and **9**.

FMOs refer to the highest occupied molecular orbital (HOMO), which donates electrons, and the lowest unoccupied molecular orbital (LUMO), which accepts electrons (Figure 7). These orbitals are essential for studying the reactivity of molecules, as they can be used to predict the electron transfer and excitation processes between orbitals based on the energy gap between HOMO and LUMO. As the energy gap widens, excitation energy rises, while reactivity falls (Tanaka, 2024).

Several physical parameters can be calculated from FMOs using the same computational method and basis sets. These parameters include the energy gap [(Δ*E*) = (*E*_LUMO_–*E*_HOMO_)], hardness [(*η*) = (*E*_LUMO_–*E*_HOMO_)/2], softness [(*S*) = 1/(*E*_LUMO_–*E*_HOMO_)], electronegativity [(*X*) = (–*E*_HOMO_–*E*_LUMO_)/2], electronic chemical potential [(***μ***) = –*X*], and fractional number of electrons transferred [(**∆*N***) = - ***μ*/*η***]. The results of FMO reactivity descriptors are summarized in Table 8.

**TABLE 8:** FMO energies (eV), energy difference (eV), and corresponding parameters of investigated compounds.

Compound	E_HOMO_	E_LUMO_	ΔE	η	S = 1/η	X	μ = - X	∆N = -μ/η
5a	−0.2423	−0.0909	0.1515	0.0757	13.2031	0.1666	−0.1666	2.1995
5b	−0.2239	−0.1157	0.1082	0.0541	18.4860	0.1698	−0.1698	3.1381
7a	−0.2260	−0.1008	0.1253	0.0626	15.9681	0.1634	−0.1634	2.6094
7b	−0.2232	−0.0900	0.1332	0.0666	15.0161	0.1566	−0.1566	2.3519
9	−0.2329	−0.1256	0.1073	0.0537	18.6393	0.1793	−0.1793	3.3418

Softness indicates the sensitivity of the π-electron cloud of a molecule to potential disruptions from chemical processes. Consequently, the electronic characteristics of terminal substituents may influence softness. (Ahmed et al., 2020). For example, compounds **5b** and **9** exhibited higher softness values owing to the presence of the COOH group in **5b** and high molecular weight of compound **9**. Moreover, the electronegativity and fractional number of electrons transferred were greater in **9** than those in the other compounds, attributable to the higher electron density in this compound.

## 3 Materials and methods

### 3.1 Materials and equipment

Details regarding the materials and equipment characterization are provided in the Supplementary Material.

### 3.2 General procedure for condensation

A mixture containing 2-(2-isopropyl-5-methylphenoxy)acetohydrazide (**3**) (0.01 mol) and different acid anhydrides (0.01 mol), i.e., phthalic anhydride (**4a**), 1,2,4-benzene tricarboxylic acid anhydride (**4b**), 1,8-naphthalic anhydride (**6a**), 4-amino-1,8-naphthalic anhydride (**6b**), and pyromellitic dianhydride (**8**) in 15 mL DMF and a few drops of glacial acetic acid was refluxed for 4–6 h. The excess solvent was removed under reduced pressure, and the residue was poured into cold water (200 mL). The obtained solid was filtered and crystallized from ethanol to yield compounds **5a**, **5b**, **7a**, **7b**, and **9**.

#### 3.2.1 *N*-(1,3-dioxoisoindolin-2-yl)-2-(2-isopropyl-5-methylphenoxy)acetamide (5a)

Compound **5a** as white crystals (79% yield); *R*_f_ = 0.44 (n-hexane:ethyl acetate, 1:2, V/V); m.p = 148°C–150°C; IR(KBr) *ν*_max_ (cm^−1^): 3427 (NH), 2955 (CH), 1792, 1744 (C=O, conjugated anhydride) observed as strong bands; ^1^H-NMR (500 MHz, DMSO-*d*_6_) *δ*_H_: 10.86 (s, 1H, **NH**), 7.95 (d, *J* = 2.5 Hz, 2H, Ar-H), 7.91 (d, *J* = 3.0 Hz, 2H, Ar-H), 7.07 (d, *J* = 7.5 Hz, 1H, Ar-H), 6.79 (s, 1H, Ar-H), 6.75 (d, *J* = 7.0 Hz, 1H, Ar-H), 4.80 (s, 2H, **CH**_**2**_), 3.23 (m, 1H, **CH**), 2.27 (s, 3H, **CH**_**3**_), 1.13 (d, *J* = 6.5 Hz, 6H, **CH**_**3**_-CH-**CH**_**3**_); ^13^C-NMR (125 MHz, DMSO- *d*_6_) *δ*_C_: 168.4, 165.5, 156.3 (C=O, amide), 136.5, 135.9, 134.1, 129.9, 126.3, 124.3, 122.6, 113.7 (Ar-**C**), 66.8 (OCH_2_), 26.2, 23.3, 21.4; Anal. calculated for C_20_H_20_N_2_O_4_: C, 68.17; H, 5.72; N, 7.95; Found: C, 67.94; H, 5.59; N, 8.02.

#### 3.2.2 2-(2-(2-Isopropyl-5-methylphenoxy)acetamido)-1,3-dioxoisoindoline-5-carboxylic acid (5b)

Compound **5b** as yellowish-white crystals (80% yield); *R*_f_ = 0.48 (n-hexane:ethyl acetate, 1:3, V/V); m.p = 190°C–192°C; IR(KBr) *ν*_max_ (cm^−1^): 3358 (NH), 3420 cm^-1^ (OH), 1773, 1732 (C=O, conjugated anhydride) observed as strong bands; ^1^H-NMR (500 MHz, DMSO-*d*_6_) *δ*_H_: 10.99 (bs, 1H, **COOH**), 10.13 (bs, 1H, **NH**), 8.41 (d, *J* = 3.0 Hz, 1H, Ar-H), 8.32 (d, *J* = 1.5 Hz, 1H, Ar-H), 8.07 (s, 1H, Ar-H), 7.42–6.91 (m, 1H, Ar-H), 6.74–6.28 (m, 2H, Ar-H), 4.80 (m, 2H, **CH**_**2**_), 3.31 (m, 1H, **CH**), 2.23 (s, 3H, **CH**_**3**_), 1.13 (s, 6H, **CH**_**3**_-CH-**CH**_**3**_); ^13^C-NMR (125 MHz, DMSO-*d*_6_) *δ*_C_: 168.4 (COOH), 167.6, 164.8, 155.4 (C=O, amide), 136.5, 136.4, 134.1, 130.3, 126.2, 126.1, 124.8, 124.3, 122.6, 122.2, 113.6, 113.4 (Ar-**C**), 66.8 (OCH_2_), 26.2, 23.3, 21.4; Anal. calculated for C_21_H_20_N_2_O_6_: C, 63.63; H, 5.09; N, 7.07; Found: C, 63.52; H, 4.97; N, 7.19.

#### 3.2.3 *N*-(1,3-dioxo-*1H*-benzo[*de*]isoquinolin-2(*3H*)-yl)-2-(2-isopropyl-5-methylphenoxy) acetamide (7a)

Compound **7a** as off-white crystals (82% yield); *R*_f_ = 0.52 (n-hexane:ethyl acetate, 1:3, V/V); m.p = 198°C–200°C; IR(KBr) *ν*_max_ (cm^−1^): 3191 (NH), 2961 (CH), 1773, 1732 (C=O, conjugated anhydride) observed as strong bands; ^1^H-NMR (500 MHz, DMSO-*d*_6_) *δ*_H_: 10.89 (s, 1H, **NH**), 8.52 (d, *J* = 7.5 Hz, 2H, Ar-H), 8.48 (d, *J* = 7.5 Hz, 2H, Ar-H), 7.87 (t, *J* = 7.5 Hz, 2H, Ar-H), 7.06 (d, *J* = 7.5 Hz, 1H, Ar-H), 6.90 (s, 1H, Ar-H), 6.75 (d, *J* = 7.5 Hz, 1H, Ar-H), 4.81 (s, 2H, **CH**_**2**_), 3.34 (m, 1H, **CH**), 2.30 (s, 3H, **CH**_**3**_), 1.14 (d, *J* = 7.0 Hz, 6H, **CH**_**3**_-CH-**CH**_**3**_); ^13^C-NMR (125 MHz, DMSO-*d*_6_) *δ*_C_: 167.8, 162.1, 155.4 (C=O, amide), 136.5, 135.9, 135.8, 134.1, 132.9, 132.1, 128.1, 127.9, 127.7, 126.2, 122.5, 122.1, 113.8 (Ar-**C**), 66.9 (OCH_2_), 26.2, 23.3, 21.5; Anal. calculated for C_24_H_22_N_2_O_4_: C, 71.63; H, 5.51; N, 6.96; Found: C, 71.54; H, 5.42; N, 7.01.

#### 3.2.4 *N*-(6-amino-1,3-dioxo-*1H*-benzo[*de*]isoquinolin-2(*3H*)-yl)-2-(2-isopropyl-5-methylphenoxy)acetamide (7b)

Compound **7b** as pale yellow crystals (84% yield); *R*_f_ = 0.54 (n-hexane:ethyl acetate, 1:3, V/V); m.p = 290°C–292°C; IR(KBr) *ν*_max_ (cm^−1^): 3436, 3350 (NH_2_), 3191 (NH), 1740, 1,698 (C=O, conjugated anhydride) observed as strong bands; ^1^H-NMR (500 MHz, DMSO-*d*_6_) *δ*_H_: 10.09 (s, 1H, **NH**), 8.61 (d, *J* = 8.5 Hz, 1H, Ar-H), 8.35 (d, *J* = 7.0 Hz, 1H, Ar-H), 8.11 (d, *J* = 7.5 Hz, 1H, Ar-H), 7.71 (s, 2H, **NH**_**2**_), 7.60 (t, *J* = 7.0 Hz, 1H, Ar-H), 7.01 (d, *J* = 7.0 Hz, 1H, Ar-H), 6.81 (d, *J* = 7.5 Hz, 2H, Ar-H), 6.71 (s, 1H, Ar-H), 4.54 (s, 2H, **CH**_**2**_), 2.84 (m, 1H, **CH**), 2.19 (s, 3H, **CH**_**3**_), 1.10 (s, 6H, **CH**_**3**_-CH-**CH**_**3**_); ^13^C-NMR (125 MHz, DMSO-*d*_6_) *δ*_C_: 162.5, 160.8, 154.4 (C=O, amide), 136.4, 134.1, 133.5, 133.0, 131.3, 131.2, 126.1, 124.8, 122.3, 119.7, 118.7, 113.4, 109.2 (Ar-**C**), 102.6 (CNH_2_), 66.8 (OCH_2_), 26.1, 23.3, 21.4; Anal. calculated for C_24_H_23_N_3_O_4_: C, 69.05; H, 5.55; N, 10.07; Found: C, 68.85; H, 5.43; N, 10.19.

#### 3.2.5 *N,N'*-(1,3,5,7-tetraoxopyrrolo[3,4-*f*]isoindole-2,6(*1H,3H,5H,7H*)-diyl)bis(2-(2-isopropyl-5-methylphenoxy)acetamide) (9)

Compound **9** as yellowish-white crystals (85% yield); *R*_f_ = 0.44 (n-hexane:ethyl acetate, 1:3, V/V); m.p > 300°C; IR(KBr) *ν*_max_ (cm^−1^): 3358 (NH), 2962 (CH), 1744, 1715 (C=O, conjugated anhydride) observed as strong bands; ^1^H-NMR (500 MHz, DMSO-*d*_6_) *δ*_H_: 11.96 (s, 1H, **NH**), 11.03 (s, 1H, **NH**), 8.45 (s, 1H, pyromellitic-H), 8.29 (s, 1H, pyromellitic-H), 7.07–6.59 (m, 6H, Ar-H), 4.82 (s, 4H, 2**CH**_**2**_), 3.07 (m, 2H, 2**CH**), 2.27 (s, 6H, 2**CH**_**3**_), 1.13 (d, *J* = 6.5 Hz, 12H, 2**CH**_**3**_-CH-**CH**_**3**_); ^13^C-NMR (125 MHz, DMSO-*d*_6_) *δ*_C_: 168.3, 166.8, 163.9, 163.8, 155.2 (C=O, amide), 138.4, 136.5, 135.7, 135.6, 134.9, 134.1, 126.3, 122.6, 119.7, 118.5, 118.4, 113.6, 113.5 (Ar-**C**), 66.7, 66.6 (OCH_2_), 26.3, 24.8, 23.3, 23.2, 21.5, 21.3; Anal. calculated for C_34_H_34_N_4_O_8_: C, 65.17; H, 5.47; N, 8.94; Found: C, 65.02; H, 5.34; N, 9.01.

### 3.3 *In silico* physicochemical and pharmacokinetic predictions

The method of ADMET study is provided in the Supplementary Material.

### 3.4 *In silico* molecular docking

The method of *In silico* Molecular Docking study is provided in the Supplementary Material.

### 3.5 Biological evaluation

#### 3.5.1 Hemolytic activity assay

The hemolytic effect of different compounds was evaluated using an existing method. (Diaconu et al., 2020). One milliliter of rat blood was collected, added to a sterile, screw-top EDTA tube, and centrifuged at 3000 rpm for 20 min. The upper layer was decanted, and the erythrocytes were rinsed several times with 10 mL of cooled isotonic and sterile phosphate-buffered saline (PBS) with a pH of 7.4. The rinsed cells were re-suspended in 20 mL sterile, cold PBS. The compounds (serial dilutions of 62.5–1,000 μg/mL in DMSO) were added to the erythrocyte solution and incubated for 60 min at 37°C. After centrifugation at 3000 rpm for 10 min, the absorbance of hemoglobin in the supernatant at 540 nm was used to calculate the hemolysis rate. Triton X-100 (0.1% in PBS) was used as a positive control, and the vehicle (PBS/DMSO) was used as the negative control. The hemolysis percentage was calculated as follows: Hemolysis percentage = (Ab of sample − Ab of negative control)/(Ab of positive control–Ab of negative control) × 100.

#### 3.5.2 Drugs

NTZ was orally administered as a reference drug at a dose of 250 mg/kg/d for 10 successive days, starting from the 7th day of infection. (Fahmy et al., 2021).

#### 3.5.3 Preparation of *Cryptosporidium* oocysts

*Cryptosporidium* oocysts were obtained from the Parasitology Lab, Theodor Bilharz Research Institute, Giza, Egypt. Before infection, the oocysts were concentrated and counted in PBS using a hemocytometer. To maintain the organism cycle, five mice were inoculated with 3000–3500 oocysts using a tuberculin syringe in an intra-esophageal manner. (Operario et al., 2015).

#### 3.5.4 Experimental animals

Eighty healthy laboratory-bred adult male Swiss albino mice weighing 30–40 g were purchased from the animal house of the Faculty of Pharmacy, Pharos University. The animals were allowed to acclimatize for 1 week to the environmental conditions and housed in grouped plastic cages away from direct sunlight, under appropriate sanitary conditions and controlled temperature and humidity.

#### 3.5.5 Immunosuppression

Immune suppression was induced and maintained throughout the experiment by administering dexamethasone (Dexazone, Al Kahira Pharmaceutical and Chemical Industries) through oral-gastric gavage at a dose of 0.25 mg/g/d for 14 consecutive days prior to inoculation with *Cryptosporidium* oocysts. (Najoom et al., 2021).

#### 3.5.6 Infection

On day 15 of dexamethasone treatment, mice in the infected groups were inoculated with 10^4^ *Cryptosporidium* oocysts using oral-gastric gavage. Before infection, oocysts were concentrated and counted in PBS using a hemocytometer.

#### 3.5.7 Study design

Mice were distributed equally into eight groups of 10 each. At the end of the experiment, all animals were anesthetized for parasitological, ultrastructural, immunological, and biochemical studies. Mice grouping details are provided in the Supplementary Material.

#### 3.5.8 Parasitological examination

On the final day of the experiment (30 days post infection), fresh fecal pellets from the infected mice in each group were collected to count the number of oocysts. Every sample was homogenized through suspension in double-distilled H_2_O. Subsequently, a fecal smear of 1 mg feces was prepared and stained using the modified Ziehl–Neelsen staining method. The stained fecal smear was examined microscopically, and the *Cryptosporidium* oocysts were counted. The number of oocysts per mg feces for each mouse was tabulated as the mean value for each group. All reported data represent the means of three independent trials.

#### 3.5.9 Ultrastructural analysis

##### 3.5.9.1 SEM

Fecal samples from each group were harvested in a 2.5% glutaraldehyde solution and examined using a scanning electron microscope (Hassan et al., 2023) to detect morphological changes. An aliquot (20 μL) of dispersed flow cell biofilm sample was fixed in an equal volume of 5.0% glutaraldehyde in sterile 1× PBS. The samples were immobilized and attached to 0.01% poly-L-lysine (Sigma, USA) coated round coverslips (12 mm) and subjected to high-resolution imaging using an in-lens secondary electron detector at an accelerating voltage of 3 kV (Zeiss 55 VP field-emission SEM).

##### 3.5.9.2 TEM

The small intestine of each mouse was removed, cut into small pieces (1 mm^3^), fixed in 2% glutaraldehyde in 0.1 M phosphate buffer, postfixed in 1% osmium tetraoxide for 2 h at 4°C, dehydrated, and embedded in Epon. Semi-thin and ultrathin sections (two samples per mouse) were prepared using an ultramicrotome. The sections were stained and analyzed using a transmission electron microscope.

##### 3.5.9.3 Biochemical measurements

Blood samples were collected from each group, transferred into tubes, and centrifuged at 3000 rpm for 5 min. The clear, non-hemolyzed supernatant serum was separated into clean tubes and stored at −20°C until use. Alanine aminotransferase (ALT) and aspartate aminotransferase (AST) levels were determined using commercially available spectrophotometric diagnostic kits (Sigma-Aldrich) according to the manufacturer instructions.

##### 3.5.9.4 Immunological measurements

Serum concentrations of IFN-γ, TNF-α, IL-6, and IL-10 for each group were determined using an enzyme-linked immunosorbent assay reader, following the manufacturer’s protocol.

##### 3.5.9.5 Statistical analysis

The results were calculated, tabulated, and statistically analyzed using the statistical computer program SPSS version 24 (Windows 10). Data were expressed as mean ± standard deviation (SD). Differences between groups were determined using a one-way analysis of variance (ANOVA) to compare one variable across groups. The level of significance was defined as *P* < 0.05.

##### 3.5.9.6 Ethics approval

The experiments were performed in accordance with the guidelines of the Institutional Animal Care and Use Committee (IACUC) at the Faculty of Medicine, Alexandria University (AU 04/24/12/30/3/03) as well as the Animal Research: Reporting of *In Vivo* Experiments (ARRIVE) guidelines. The mice anesthetized through intraperitoneal injections of ketamine hydrochloride (100 mg/kg) and xylazine (10 mg/kg).

## 5 Conclusion

This study reports the design and synthesis of novel phenoxy acetamide derivatives (**5a**, **5b**, **7a**, **7b**, and **9**) based on a thymol moiety for target parasitological treatments. All synthesized compounds demonstrated a reduction in oocyst counts, with compound **7b** exhibiting the highest percentage reduction (67%). Moreover, all treated groups showed significant changes in the biochemical parameter levels compared with the positive control group. The mean serum levels of INF-γ, IL-6, and IL-10 were significantly reduced in all treated groups compared with the infected control group. DFT calculations indicated that compounds **5a**, **5b**, and **7b** exhibited a high energy gap between FMOs and high dipole moment values, which may be strongly correlated with their bioactivity. Therefore, compounds **5a**, **5b**, and **7b** represent promising candidates for the development of efficient antiparasitic drugs.

## Data Availability

The raw data supporting the conclusions of this article will be made available by the authors, without undue reservation.
