# Supplementary material for: Synthesis and characterization of thymol-derived phenoxy acetamide derivatives using DFT, molecular docking, and parasitological investigations
Source: Front Chem. 2025 May 21;13:1579923. doi: 10.3389/fchem.2025.1579923 (PMC12134901; doi:10.3389/fchem.2025.1579923)
Supplement: Supplementary file 1 [file DataSheet1.docx]

**Supplementary information**

| **Serial** | **content** | **page** |
| --- | --- | --- |
| **1.** | Copy of NMR spectra |  |
|  | Figure S1. ^1^HNMR of **5a** | **S2** |
|  | Figure S2. ^13^C NMR of **5a**  Figure S3. FT-IR of compound **5a** | **S3**  **S4** |
|  | Figure S4. ^1^HNMR of **5b** | **S5** |
|  | Figure S5. ^13^C NMR of **5b**  Figure S6. FT-IR of compound **5b** | **S6**  **S7** |
|  | Figure S7. ^1^HNMR of **7a** | **S8** |
|  | Figure S8. ^13^C NMR of **7a** | **S9** |
|  | Figure S9. FT-IR of compound **7a**  Figure S7. ^1^HNMR of **7b**  Figure S5. ^13^C NMR of **7b**  Figure S3. FT-IR of compound **7b**  Figure S7. ^1^HNMR of **9**  Figure S5. ^13^C NMR of **9**  Figure S3. FT-IR of compound **9**  **3.2.** Chemistry **3.1.** Equipment and analytical techniques  **3.3.7.** Study design: **Table 1.** Mice grouping details | **S10**  **S11**  **S12**  **S13**  **S14**  **S15**  **S16**  **S17**  **S18**  **S19** |

**

** **Figure S1.** ^1^H NMR of compound **5a** (DMSO-d6)

**

** **Figure S2**. ^13^C NMR of compound **5a** (DMSO-d6)

**Figure S3**. FT-IR of compound **5a**

**

**

**Figure S4.** ^1^H NMR of compound **5b** (DMSO-d6)

**

** **Figure S5**. ^13^C NMR of compound **5b** (DMSO-d6)

**Figure S6**. FT-IR of compound **5b**

**

**

**Figure S7.** ^1^H NMR of compound **7a** (DMSO-d6)

**

**

**Figure S8**. ^13^C NMR of compound **7a** (DMSO-d6)

**Figure S9**. FT-IR of compound **7a**

**

**

**Figure S10.** ^1^H NMR of compound **7b** (DMSO-d6)

**

**

**Figure S11**. ^13^C NMR of compound **7b** (DMSO-d6)

**Figure S12**. FT-IR of compound **7b**

**

**

**Figure S13.** ^1^H NMR of compound **9** (DMSO-d6)

**

Figure S14**. ^13^C NMR of compound **9** (DMSO-d6)

**Figure S15**. FT-IR of compound **9**

**Material and method**

**3.1. Equipment and analytical techniques**

Commercially available solvents and reagents were purified according to reported standard procedures. All melting points are measured on Melt-Temp apparatus Model number 1002D, 220-240 V, 200 watts, 50/60 Hz and are uncorrected. Thin layer chromatography (TLC) was performed on aluminum plates silica gel (Fluka, 60 F254 layer thickness 0.2 mm). Visualization of the TLC during monitoring of the reaction was done by UV VILBER LOURMAT 4w-365 nm or 254 nm tube. NMR spectra measured with JEOLJNM-ECA 500 MHz spectrometer, using (DMSO-d6) solvent. Chemical shifts (δ) are given in ppm relative to the signal for TMS as internal standard, and coupling constants are quoted in Hz. 13C NMR spectra were recorded on a 100 MHz and 125 MHz spectrometer. The IR spectra were obtained using a Perkin-Elmer FT-IR spectrum BX and Bruker tensor 37 FT-IR, and wavelengths are reported in Cm^-1^.

**3.2. Chemistry**

**General procedure of condensation**

A mixture of 2-(2-isopropyl-5-methylphenoxy)acetohydrazide (**3**) (0.01 mol) with different acid anhydride (0.01 mol) namely phthalic anhydride (**4a**), 1,2,4-benzene tricarboxylic acid anhydride (**4b**), 1,8-naphthalic anhydride (**6a**), 4-amino-1,8-naphthalic anhydride (**6b**)and pyromellitic dianhydride (**8**) in 15 ml DMF and drops of glacial acetic acid was refluxed for 4-6 hrs. the excess solvent was removed under reduced pressure then the residue was poured into cold water (200 ml). The obtained solid was filtered off and crystallized from ethanol to afford compounds **5a, 5b, 7a, 7b and 9**; respectively.

**3.2.1.** *N*-(1,3-Dioxoisoindolin-2-yl)-2-(2-isopropyl-5-methylphenoxy)acetamide (**5a**)

Compound **5a** as white crystals (79% yield); R_f_ = 0.44 ( n-Hexane : ethyl acetate, 1:2, V/V); m.p = 148-150 ºC; IR(KBr) ν_max_ (cm^−1^): 3427 (NH), 2955 (CH), 1792, 1744 (C=O, conjugated anhydride) were observed as strong bands; ^1^H NMR (500 MHz, DMSO-d6) δ_H_: 10.86 (s, 1H, **NH**), 7.95 (d, *J* = 2.5 Hz, 2H, Ar-H), 7.91 (d, *J* = 3.0 Hz, 2H, Ar-H), 7.07 (d, *J* = 7.5 Hz, 1H, Ar-H), 6.79 (s, 1H, Ar-H), 6.75 (d, *J* = 7.0 Hz, 1H, Ar-H), 4.80 (s, 2H, **CH_2_**), 3.23 (m, 1H, **CH**), 2.27 (s, 3H, **CH_3_**), 1.13 (d, *J* = 6.5 Hz, 6H, **CH_3_**-CH-**CH_3_**); ^13^C NMR (125 MHz, DMSO-d6) δ_C_: 168.4, 165.5, 156.3 (C=O, amide), 136.5, 135.9, 134.1, 129.9, 126.3, 124.3, 122.6, 113.7 (Ar-**C**), 66.8 (OCH_2_), 26.2, 23.3, 21.4; Anal. calculated for C_20_H_20_N_2_O_4_: C, 68.17; H, 5.72; N, 7.95; Found: C, 67.94; H, 5.59; N, 8.02

**3.2.2.** 2-(2-(2-Isopropyl-5-methylphenoxy)acetamido)-1,3-dioxoisoindoline-5-carboxylic acid (**5b**)

Compound **5b** as yellowish-white crystals (80% yield); R_f_ = 0.48 ( n-Hexane : ethyl acetate, 1:3, V/V) ; m.p = 190-192ºC; IR(KBr) ν_max_ (cm^−1^): 3358 (NH), 3420 cm^-1^ (OH), 1773, 1732 (C=O, conjugated anhydride) were observed as strong bands; ^1^H NMR (500 MHz, DMSO-d6) δ_H_: 10.99 (bs, 1H, **COOH**), 10.13 (bs, 1H, **NH**), 8.41 (d, *J* = 3.0 Hz, 1H, Ar-H), 8.32 (d, *J* = 1.5 Hz, 1H, Ar-H), 8.07 (s, 1H, Ar-H), 7.42-6.91 (m, 1H, Ar-H), 6.74-6.28 (m, 2H, Ar-H), 4.80 (m, 2H, **CH_2_**), 3.31 (m, 1H, **CH**), 2.23 (s, 3H, **CH_3_**), 1.13 (s, 6H, **CH_3_**-CH-**CH_3_**); ^13^C NMR (125 MHz, DMSO-d6) δ_C_: 168.4 (COOH), 167.6, 164.8, 155.4 (C=O, amide), 136.5, 136.4, 134.1, 130.3, 126.2, 126.1, 124.8, 124.3, 122.6, 122.2, 113.6, 113.4 (Ar-**C**), 66.8 (OCH_2_), 26.2, 23.3, 21.4; Anal. calculated for C_21_H_20_N_2_O_6_: C, 63.63; H, 5.09; N, 7.07; Found: C, 63.52; H, 4.97; N, 7.19

**3.2.3.** *N*-(1,3-Dioxo-*1H*-benzo[*de*]isoquinolin-2(*3H*)-yl)-2-(2-isopropyl-5-methylphenoxy) acetamide (**7a**)

Compound **7a** as off-white crystals (82% yield); R_f_ = 0.52 ( n-Hexane : ethyl acetate, 1:3, V/V) ; m.p = 198-200 ºC; IR(KBr) ν_max_ (cm^−1^): 3191 (NH), 2961 (CH), 1773, 1732 (C=O, conjugated anhydride) were observed as strong bands; ^1^H NMR (500 MHz, DMSO-d6) δ_H_: 10.89 (s, 1H, **NH**), 8.52 (d, *J* = 7.5 Hz, 2H, Ar-H), 8.48 (d, *J* = 7.5 Hz, 2H, Ar-H), 7.87 (t, *J* = 7.5 Hz, 2H, Ar-H), 7.06 (d, *J* = 7.5 Hz, 1H, Ar-H), 6.90 (s, 1H, Ar-H), 6.75 (d, *J* = 7.5 Hz, 1H, Ar-H), 4.81 (s, 2H, **CH_2_**), 3.34 (m, 1H, **CH**), 2.30 (s, 3H, **CH_3_**), 1.14 (d, *J* = 7.0 Hz, 6H, **CH_3_**-CH-**CH_3_**); ^13^C NMR (125 MHz, DMSO-d6) δ_C_: 167.8, 162.1, 155.4 (C=O, amide), 136.5, 135.9, 135.8, 134.1, 132.9, 132.1, 128.1, 127.9, 127.7, 126.2, 122.5, 122.1, 113.8 (Ar-**C**), 66.9 (OCH_2_), 26.2, 23.3, 21.5; Anal. calculated for C_24_H_22_N_2_O_4_: C, 71.63; H, 5.51; N, 6.96; Found: C, 71.54; H, 5.42; N, 7.01.

**3.2.4.** *N*-(6-Amino-1,3-dioxo-*1H*-benzo[*de*]isoquinolin-2(*3H*)-yl)-2-(2-isopropyl-5-methylphenoxy)acetamide (**7b**)

Compound **7b** as pale yellow crystals (84% yield); R_f_ = 0.54 ( n-Hexane : ethyl acetate, 1:3, V/V); m.p = 290-292 ºC; IR(KBr) ν_max_ (cm^−1^): 3436, 3350 (NH_2_), 3191 (NH), 1740, 1698 (C=O, conjugated anhydride) were observed as strong bands; ^1^H NMR (500 MHz, DMSO-d6) δ_H_: 10.09 (s, 1H, **NH**), 8.61 (d, *J* = 8.5 Hz, 1H, Ar-H), 8.35 (d, *J* = 7.0 Hz, 1H, Ar-H), 8.11 (d, *J* = 7.5 Hz, 1H, Ar-H), 7.71 (s, 2H, **NH_2_**), 7.60 (t, *J* = 7.0 Hz, 1H, Ar-H), 7.01 (d, *J* = 7.0 Hz, 1H, Ar-H), 6.81 (d, *J* = 7.5 Hz, 2H, Ar-H), 6.71 (s, 1H, Ar-H), 4.54 (s, 2H, **CH_2_**), 2.84 (m, 1H, **CH**), 2.19 (s, 3H, **CH_3_**), 1.10 (s, 6H, **CH_3_**-CH-**CH_3_**); ^13^C NMR (125 MHz, DMSO-d6) δ_C_: 162.5, 160.8, 154.4 (C=O, amide), 136.4, 134.1, 133.5, 133.0, 131.3, 131.2, 126.1, 124.8, 122.3, 119.7, 118.7, 113.4, 109.2 (Ar-**C**), 102.6 (CNH_2_), 66.8 (OCH_2_), 26.1, 23.3, 21.4; Anal. calculated for C_24_H_23_N_3_O_4_: C, 69.05; H, 5.55; N, 10.07; Found: C, 68.85; H, 5.43; N, 10.19

**3.2.5.** *N,N'*-(1,3,5,7-Tetraoxopyrrolo[3,4-*f*]isoindole-2,6(*1H,3H,5H,7H*)-diyl)bis(2-(2-isopropyl-5-methylphenoxy)acetamide) (**9**)

Compound **9** as yellowish-white crystals (85% yield); R_f_ = 0.44 ( n-Hexane : ethyl acetate, 1:3, V/V); m.p > 300 ºC; IR(KBr) ν_max_ (cm^−1^): 3358(NH), 2962 (CH), 1744, 1715(C=O, conjugated anhydride) were observed as strong bands; ^1^H NMR (500 MHz, DMSO-d6) δ_H_: 11.96 (s, 1H, **NH**), 11.03 (s, 1H, **NH**), 8.45 (s, 1H, pyromellitic-H), 8.29 (s, 1H, pyromellitic-H), 7.07-6.59 (m, 6H, Ar-H), 4.82 (s, 4H, 2**CH_2_**), 3.07 (m, 2H, 2**CH**), 2.27 (s, 6H, 2**CH_3_**), 1.13 (d, *J* = 6.5 Hz, 12H, 2**CH_3_**-CH-**CH_3_**); ^13^C NMR (125 MHz, DMSO-d6) δ_C_: 168.3, 166.8, 163.9, 163.8, 155.2 (C=O, amide), 138.4, 136.5, 135.7, 135.6, 134.9, 134.1, 126.3, 122.6, 119.7, 118.5, 118.4, 113.6, 113.5 (Ar-**C**), 66.7, 66.6 (OCH_2_), 26.3, 24.8, 23.3, 23.2, 21.5, 21.3; Anal. calculated for C_34_H_34_N_4_O_8_: C, 65.17; H, 5.47; N, 8.94; Found: C, 65.02; H, 5.34; N, 9.01

| Groups | No. | Characteristics |
| --- | --- | --- |
| Group 1 | 10 | Negative control (Non infected and non-treated). |
| Group 2 | 10 | Positive control (infected and non-treated). |
| Group 3 | 10 | **NTZ** treated immunosuppressed infected mice, received NTZ (250 mg/kg/day) orally on the 7^th^ days post-infection for 10 days. |
| Group 4 | 10 | Compound **5a** immunosuppressed infected mice,  received (100 mg/kg/day) orally on the 7^th^ days post-infection for 10 days. |
| Group 5 | 10 | Compound **5b** immunosuppressed infected mice,  received (100 mg/kg/day) orally on the 7^th^ days post-infection for 10 days. |
| Group 6 | 10 | Compound **7a** treated immunosuppressed infected mice,  received (100 mg/kg/day) orally on the 7^th^ days post-infection for 10 days. |
| Group 7 | 10 | Compound **7b** treated immunosuppressed infected mice,  received (100 mg/kg/day) orally on the 7^th^ days post-infection for 10 days. |
| Group 8 | 10 | Compound **9** treated immunosuppressed infected mice,  received (100 mg/kg/day) orally on the 7^th^ days post-infection for 10 days. |

**3.3.7. Study design:**

**Table 1.** Mice grouping details

- - 1. **ADMET Analysis**

The ADMET (Absorption, Distribution, Metabolism, Excretion, and Toxicity) properties of the synthesized compounds were analyzed *in silico* using the Swiss ADME web tool (<http://www.swissadme.ch/>), a widely used platform for predicting pharmacokinetic and physicochemical properties of small molecules. The chemical structures of the compounds were drawn and converted into Simplified Molecular Input Line Entry System (SMILES) format using Chem Draw (PerkinElmer Informatics). These SMILES representations were then uploaded to the Swiss ADME interface for analysis. The tool was used to evaluate key pharmacokinetic properties, including gastrointestinal (GI) absorption, blood-brain barrier (BBB) permeability, P-glycoprotein (P-gp) substrate status, and cytochrome P450 (CYP) enzyme inhibition. Drug-likeness was assessed based on Lipinski’s rule of five, while the bioavailability score was calculated to estimate oral absorption potential. Additionally, synthetic accessibility and lead-likeness violations were analyzed to assess the feasibility of further optimization. Physicochemical properties, including molecular weight (MW), number of heavy atoms, topological polar surface area (TPSA), fraction of sp3 carbons (Csp3), hydrogen bond donors and acceptors, rotatable bonds, and molecular refractivity (MR) were predicted to understand the compounds’ structural and functional attributes. Lipophilicity was assessed using the consensus Log Po/w, while water solubility was predicted using the ESOL method, with solubility classes assigned based on the calculated Log S values [[1](#_ENREF_1)].

**3.3.11. Molecular Docking**

Molecular docking studies were conducted to explore the binding potential and interaction profiles of five synthetic compounds (5a, 5b, 7a, 7b, and 9)c with calcium-dependent protein kinase 1 (CDPK1) from *Cryptosporidium parvum* [[2](#_ENREF_2)]. The protein structure (PDB ID: 2WEI) was retrieved from the Protein Data Bank [[3](#_ENREF_3)]. The protein structure was prepared by using the geometry optimization tool in Avogadro with the MMFF94 force field. Docking simulations were performed using Auto Dock 4.2 [[4](#_ENREF_4)]. The Simulated Annealing method was utilized for docking with the default parameters, using 100 runs and 150 cycles per ligand. To validate the docking protocol, the co-crystallized of reference ligand 1-tert-butyl-3-(3-methylbenzyl)-1H-pyrazolo[3,4-d]pyrimidin-4-amine (VGG) was redocked into the active site of CDPK1. The root means square deviation (RMSD) between the redocked pose and the crystallized conformation was calculated. An RMSD value below 2 Å was considered indicative of a reliable docking protocol.

**References**

1. Bakchi, B., et al., *An overview on applications of SwissADME web tool in the design and development of anticancer, antitubercular and antimicrobial agents: A medicinal chemist's perspective.* Journal of Molecular Structure, 2022. **1259**: p. 132712.

2. Rutaganira, F.U., et al., *Inhibition of calcium dependent protein kinase 1 (CDPK1) by pyrazolopyrimidine analogs decreases establishment and reoccurrence of central nervous system disease by Toxoplasma gondii.* Journal of medicinal chemistry, 2017. **60**(24): p. 9976-9989.

3. Artz, J.D., et al., *The Cryptosporidium parvum Kinome.* BMC Genomics, 2011. **12**(1): p. 478.

4. El-Hachem, N., et al., *AutoDock and AutoDockTools for Protein-Ligand Docking: Beta-Site Amyloid Precursor Protein Cleaving Enzyme 1(BACE1) as a Case Study*, in *Neuroproteomics*, F.H. Kobeissy and S.M. Stevens, Editors. 2017, Springer New York: New York, NY. p. 391-403.

Post hoc for Cytokine levels in mice groups

| **Multiple Comparisons** | | | | | | | |
| --- | --- | --- | --- | --- | --- | --- | --- |
| Tukey HSD | | | | | | | |
| Dependent Variable | (I) G | (J) G | Mean Difference (I-J) | Std. Error | Sig. | 95% Confidence Interval | |
|  |  |  |  |  |  | Lower Bound | Upper Bound |
| TNF | G1 | G2 | -23.95000^*^ | .80401 | .000 | -26.7336 | -21.1664 |
|  |  | G3 | 1.50000 | .80401 | .590 | -1.2836 | 4.2836 |
|  |  | G4 | -3.80000^*^ | .80401 | .004 | -6.5836 | -1.0164 |
|  |  | G5 | -4.40000^*^ | .80401 | .001 | -7.1836 | -1.6164 |
|  |  | G6 | -6.60000^*^ | .80401 | .000 | -9.3836 | -3.8164 |
|  |  | G7 | -2.50000 | .80401 | .095 | -5.2836 | .2836 |
|  |  | G8 | -6.90000^*^ | .80401 | .000 | -9.6836 | -4.1164 |
|  | G2 | G1 | 23.95000^*^ | .80401 | .000 | 21.1664 | 26.7336 |
|  |  | G3 | 25.45000^*^ | .80401 | .000 | 22.6664 | 28.2336 |
|  |  | G4 | 20.15000^*^ | .80401 | .000 | 17.3664 | 22.9336 |
|  |  | G5 | 19.55000^*^ | .80401 | .000 | 16.7664 | 22.3336 |
|  |  | G6 | 17.35000^*^ | .80401 | .000 | 14.5664 | 20.1336 |
|  |  | G7 | 21.45000^*^ | .80401 | .000 | 18.6664 | 24.2336 |
|  |  | G8 | 17.05000^*^ | .80401 | .000 | 14.2664 | 19.8336 |
|  | G3 | G1 | -1.50000 | .80401 | .590 | -4.2836 | 1.2836 |
|  |  | G2 | -25.45000^*^ | .80401 | .000 | -28.2336 | -22.6664 |
|  |  | G4 | -5.30000^*^ | .80401 | .000 | -8.0836 | -2.5164 |
|  |  | G5 | -5.90000^*^ | .80401 | .000 | -8.6836 | -3.1164 |
|  |  | G6 | -8.10000^*^ | .80401 | .000 | -10.8836 | -5.3164 |
|  |  | G7 | -4.00000^*^ | .80401 | .003 | -6.7836 | -1.2164 |
|  |  | G8 | -8.40000^*^ | .80401 | .000 | -11.1836 | -5.6164 |
|  | G4 | G1 | 3.80000^*^ | .80401 | .004 | 1.0164 | 6.5836 |
|  |  | G2 | -20.15000^*^ | .80401 | .000 | -22.9336 | -17.3664 |
|  |  | G3 | 5.30000^*^ | .80401 | .000 | 2.5164 | 8.0836 |
|  |  | G5 | -.60000 | .80401 | .994 | -3.3836 | 2.1836 |
|  |  | G6 | -2.80000^*^ | .80401 | .048 | -5.5836 | -.0164 |
|  |  | G7 | 1.30000 | .80401 | .735 | -1.4836 | 4.0836 |
|  |  | G8 | -3.10000^*^ | .80401 | .024 | -5.8836 | -.3164 |
|  | G5 | G1 | 4.40000^*^ | .80401 | .001 | 1.6164 | 7.1836 |
|  |  | G2 | -19.55000^*^ | .80401 | .000 | -22.3336 | -16.7664 |
|  |  | G3 | 5.90000^*^ | .80401 | .000 | 3.1164 | 8.6836 |
|  |  | G4 | .60000 | .80401 | .994 | -2.1836 | 3.3836 |
|  |  | G6 | -2.20000 | .80401 | .181 | -4.9836 | .5836 |
|  |  | G7 | 1.90000 | .80401 | .321 | -.8836 | 4.6836 |
|  |  | G8 | -2.50000 | .80401 | .095 | -5.2836 | .2836 |
|  | G6 | G1 | 6.60000^*^ | .80401 | .000 | 3.8164 | 9.3836 |
|  |  | G2 | -17.35000^*^ | .80401 | .000 | -20.1336 | -14.5664 |
|  |  | G3 | 8.10000^*^ | .80401 | .000 | 5.3164 | 10.8836 |
|  |  | G4 | 2.80000^*^ | .80401 | .048 | .0164 | 5.5836 |
|  |  | G5 | 2.20000 | .80401 | .181 | -.5836 | 4.9836 |
|  |  | G7 | 4.10000^*^ | .80401 | .002 | 1.3164 | 6.8836 |
|  |  | G8 | -.30000 | .80401 | 1.000 | -3.0836 | 2.4836 |
|  | G7 | G1 | 2.50000 | .80401 | .095 | -.2836 | 5.2836 |
|  |  | G2 | -21.45000^*^ | .80401 | .000 | -24.2336 | -18.6664 |
|  |  | G3 | 4.00000^*^ | .80401 | .003 | 1.2164 | 6.7836 |
|  |  | G4 | -1.30000 | .80401 | .735 | -4.0836 | 1.4836 |
|  |  | G5 | -1.90000 | .80401 | .321 | -4.6836 | .8836 |
|  |  | G6 | -4.10000^*^ | .80401 | .002 | -6.8836 | -1.3164 |
|  |  | G8 | -4.40000^*^ | .80401 | .001 | -7.1836 | -1.6164 |
|  | G8 | G1 | 6.90000^*^ | .80401 | .000 | 4.1164 | 9.6836 |
|  |  | G2 | -17.05000^*^ | .80401 | .000 | -19.8336 | -14.2664 |
|  |  | G3 | 8.40000^*^ | .80401 | .000 | 5.6164 | 11.1836 |
|  |  | G4 | 3.10000^*^ | .80401 | .024 | .3164 | 5.8836 |
|  |  | G5 | 2.50000 | .80401 | .095 | -.2836 | 5.2836 |
|  |  | G6 | .30000 | .80401 | 1.000 | -2.4836 | 3.0836 |
|  |  | G7 | 4.40000^*^ | .80401 | .001 | 1.6164 | 7.1836 |
| INF | G1 | G2 | -374.90000^*^ | 2.26561 | .000 | -382.7439 | -367.0561 |
|  |  | G3 | -79.60000^*^ | 2.26561 | .000 | -87.4439 | -71.7561 |
|  |  | G4 | -135.30000^*^ | 2.26561 | .000 | -143.1439 | -127.4561 |
|  |  | G5 | -151.70000^*^ | 2.26561 | .000 | -159.5439 | -143.8561 |
|  |  | G6 | -266.30000^*^ | 2.26561 | .000 | -274.1439 | -258.4561 |
|  |  | G7 | -72.70000^*^ | 2.26561 | .000 | -80.5439 | -64.8561 |
|  |  | G8 | -191.70000^*^ | 2.26561 | .000 | -199.5439 | -183.8561 |
|  | G2 | G1 | 374.90000^*^ | 2.26561 | .000 | 367.0561 | 382.7439 |
|  |  | G3 | 295.30000^*^ | 2.26561 | .000 | 287.4561 | 303.1439 |
|  |  | G4 | 239.60000^*^ | 2.26561 | .000 | 231.7561 | 247.4439 |
|  |  | G5 | 223.20000^*^ | 2.26561 | .000 | 215.3561 | 231.0439 |
|  |  | G6 | 108.60000^*^ | 2.26561 | .000 | 100.7561 | 116.4439 |
|  |  | G7 | 302.20000^*^ | 2.26561 | .000 | 294.3561 | 310.0439 |
|  |  | G8 | 183.20000^*^ | 2.26561 | .000 | 175.3561 | 191.0439 |
|  | G3 | G1 | 79.60000^*^ | 2.26561 | .000 | 71.7561 | 87.4439 |
|  |  | G2 | -295.30000^*^ | 2.26561 | .000 | -303.1439 | -287.4561 |
|  |  | G4 | -55.70000^*^ | 2.26561 | .000 | -63.5439 | -47.8561 |
|  |  | G5 | -72.10000^*^ | 2.26561 | .000 | -79.9439 | -64.2561 |
|  |  | G6 | -186.70000^*^ | 2.26561 | .000 | -194.5439 | -178.8561 |
|  |  | G7 | 6.90000 | 2.26561 | .107 | -.9439 | 14.7439 |
|  |  | G8 | -112.10000^*^ | 2.26561 | .000 | -119.9439 | -104.2561 |
|  | G4 | G1 | 135.30000^*^ | 2.26561 | .000 | 127.4561 | 143.1439 |
|  |  | G2 | -239.60000^*^ | 2.26561 | .000 | -247.4439 | -231.7561 |
|  |  | G3 | 55.70000^*^ | 2.26561 | .000 | 47.8561 | 63.5439 |
|  |  | G5 | -16.40000^*^ | 2.26561 | .000 | -24.2439 | -8.5561 |
|  |  | G6 | -131.00000^*^ | 2.26561 | .000 | -138.8439 | -123.1561 |
|  |  | G7 | 62.60000^*^ | 2.26561 | .000 | 54.7561 | 70.4439 |
|  |  | G8 | -56.40000^*^ | 2.26561 | .000 | -64.2439 | -48.5561 |
|  | G5 | G1 | 151.70000^*^ | 2.26561 | .000 | 143.8561 | 159.5439 |
|  |  | G2 | -223.20000^*^ | 2.26561 | .000 | -231.0439 | -215.3561 |
|  |  | G3 | 72.10000^*^ | 2.26561 | .000 | 64.2561 | 79.9439 |
|  |  | G4 | 16.40000^*^ | 2.26561 | .000 | 8.5561 | 24.2439 |
|  |  | G6 | -114.60000^*^ | 2.26561 | .000 | -122.4439 | -106.7561 |
|  |  | G7 | 79.00000^*^ | 2.26561 | .000 | 71.1561 | 86.8439 |
|  |  | G8 | -40.00000^*^ | 2.26561 | .000 | -47.8439 | -32.1561 |
|  | G6 | G1 | 266.30000^*^ | 2.26561 | .000 | 258.4561 | 274.1439 |
|  |  | G2 | -108.60000^*^ | 2.26561 | .000 | -116.4439 | -100.7561 |
|  |  | G3 | 186.70000^*^ | 2.26561 | .000 | 178.8561 | 194.5439 |
|  |  | G4 | 131.00000^*^ | 2.26561 | .000 | 123.1561 | 138.8439 |
|  |  | G5 | 114.60000^*^ | 2.26561 | .000 | 106.7561 | 122.4439 |
|  |  | G7 | 193.60000^*^ | 2.26561 | .000 | 185.7561 | 201.4439 |
|  |  | G8 | 74.60000^*^ | 2.26561 | .000 | 66.7561 | 82.4439 |
|  | G7 | G1 | 72.70000^*^ | 2.26561 | .000 | 64.8561 | 80.5439 |
|  |  | G2 | -302.20000^*^ | 2.26561 | .000 | -310.0439 | -294.3561 |
|  |  | G3 | -6.90000 | 2.26561 | .107 | -14.7439 | .9439 |
|  |  | G4 | -62.60000^*^ | 2.26561 | .000 | -70.4439 | -54.7561 |
|  |  | G5 | -79.00000^*^ | 2.26561 | .000 | -86.8439 | -71.1561 |
|  |  | G6 | -193.60000^*^ | 2.26561 | .000 | -201.4439 | -185.7561 |
|  |  | G8 | -119.00000^*^ | 2.26561 | .000 | -126.8439 | -111.1561 |
|  | G8 | G1 | 191.70000^*^ | 2.26561 | .000 | 183.8561 | 199.5439 |
|  |  | G2 | -183.20000^*^ | 2.26561 | .000 | -191.0439 | -175.3561 |
|  |  | G3 | 112.10000^*^ | 2.26561 | .000 | 104.2561 | 119.9439 |
|  |  | G4 | 56.40000^*^ | 2.26561 | .000 | 48.5561 | 64.2439 |
|  |  | G5 | 40.00000^*^ | 2.26561 | .000 | 32.1561 | 47.8439 |
|  |  | G6 | -74.60000^*^ | 2.26561 | .000 | -82.4439 | -66.7561 |
|  |  | G7 | 119.00000^*^ | 2.26561 | .000 | 111.1561 | 126.8439 |
| IL_6 | G1 | G2 | -172.40000^*^ | 1.04759 | .000 | -176.0269 | -168.7731 |
|  |  | G3 | -23.30000^*^ | 1.04759 | .000 | -26.9269 | -19.6731 |
|  |  | G4 | -88.70000^*^ | 1.04759 | .000 | -92.3269 | -85.0731 |
|  |  | G5 | -93.60000^*^ | 1.04759 | .000 | -97.2269 | -89.9731 |
|  |  | G6 | -149.30000^*^ | 1.04759 | .000 | -152.9269 | -145.6731 |
|  |  | G7 | -55.00000^*^ | 1.04759 | .000 | -58.6269 | -51.3731 |
|  |  | G8 | -127.60000^*^ | 1.04759 | .000 | -131.2269 | -123.9731 |
|  | G2 | G1 | 172.40000^*^ | 1.04759 | .000 | 168.7731 | 176.0269 |
|  |  | G3 | 149.10000^*^ | 1.04759 | .000 | 145.4731 | 152.7269 |
|  |  | G4 | 83.70000^*^ | 1.04759 | .000 | 80.0731 | 87.3269 |
|  |  | G5 | 78.80000^*^ | 1.04759 | .000 | 75.1731 | 82.4269 |
|  |  | G6 | 23.10000^*^ | 1.04759 | .000 | 19.4731 | 26.7269 |
|  |  | G7 | 117.40000^*^ | 1.04759 | .000 | 113.7731 | 121.0269 |
|  |  | G8 | 44.80000^*^ | 1.04759 | .000 | 41.1731 | 48.4269 |
|  | G3 | G1 | 23.30000^*^ | 1.04759 | .000 | 19.6731 | 26.9269 |
|  |  | G2 | -149.10000^*^ | 1.04759 | .000 | -152.7269 | -145.4731 |
|  |  | G4 | -65.40000^*^ | 1.04759 | .000 | -69.0269 | -61.7731 |
|  |  | G5 | -70.30000^*^ | 1.04759 | .000 | -73.9269 | -66.6731 |
|  |  | G6 | -126.00000^*^ | 1.04759 | .000 | -129.6269 | -122.3731 |
|  |  | G7 | -31.70000^*^ | 1.04759 | .000 | -35.3269 | -28.0731 |
|  |  | G8 | -104.30000^*^ | 1.04759 | .000 | -107.9269 | -100.6731 |
|  | G4 | G1 | 88.70000^*^ | 1.04759 | .000 | 85.0731 | 92.3269 |
|  |  | G2 | -83.70000^*^ | 1.04759 | .000 | -87.3269 | -80.0731 |
|  |  | G3 | 65.40000^*^ | 1.04759 | .000 | 61.7731 | 69.0269 |
|  |  | G5 | -4.90000^*^ | 1.04759 | .005 | -8.5269 | -1.2731 |
|  |  | G6 | -60.60000^*^ | 1.04759 | .000 | -64.2269 | -56.9731 |
|  |  | G7 | 33.70000^*^ | 1.04759 | .000 | 30.0731 | 37.3269 |
|  |  | G8 | -38.90000^*^ | 1.04759 | .000 | -42.5269 | -35.2731 |
|  | G5 | G1 | 93.60000^*^ | 1.04759 | .000 | 89.9731 | 97.2269 |
|  |  | G2 | -78.80000^*^ | 1.04759 | .000 | -82.4269 | -75.1731 |
|  |  | G3 | 70.30000^*^ | 1.04759 | .000 | 66.6731 | 73.9269 |
|  |  | G4 | 4.90000^*^ | 1.04759 | .005 | 1.2731 | 8.5269 |
|  |  | G6 | -55.70000^*^ | 1.04759 | .000 | -59.3269 | -52.0731 |
|  |  | G7 | 38.60000^*^ | 1.04759 | .000 | 34.9731 | 42.2269 |
|  |  | G8 | -34.00000^*^ | 1.04759 | .000 | -37.6269 | -30.3731 |
|  | G6 | G1 | 149.30000^*^ | 1.04759 | .000 | 145.6731 | 152.9269 |
|  |  | G2 | -23.10000^*^ | 1.04759 | .000 | -26.7269 | -19.4731 |
|  |  | G3 | 126.00000^*^ | 1.04759 | .000 | 122.3731 | 129.6269 |
|  |  | G4 | 60.60000^*^ | 1.04759 | .000 | 56.9731 | 64.2269 |
|  |  | G5 | 55.70000^*^ | 1.04759 | .000 | 52.0731 | 59.3269 |
|  |  | G7 | 94.30000^*^ | 1.04759 | .000 | 90.6731 | 97.9269 |
|  |  | G8 | 21.70000^*^ | 1.04759 | .000 | 18.0731 | 25.3269 |
|  | G7 | G1 | 55.00000^*^ | 1.04759 | .000 | 51.3731 | 58.6269 |
|  |  | G2 | -117.40000^*^ | 1.04759 | .000 | -121.0269 | -113.7731 |
|  |  | G3 | 31.70000^*^ | 1.04759 | .000 | 28.0731 | 35.3269 |
|  |  | G4 | -33.70000^*^ | 1.04759 | .000 | -37.3269 | -30.0731 |
|  |  | G5 | -38.60000^*^ | 1.04759 | .000 | -42.2269 | -34.9731 |
|  |  | G6 | -94.30000^*^ | 1.04759 | .000 | -97.9269 | -90.6731 |
|  |  | G8 | -72.60000^*^ | 1.04759 | .000 | -76.2269 | -68.9731 |
|  | G8 | G1 | 127.60000^*^ | 1.04759 | .000 | 123.9731 | 131.2269 |
|  |  | G2 | -44.80000^*^ | 1.04759 | .000 | -48.4269 | -41.1731 |
|  |  | G3 | 104.30000^*^ | 1.04759 | .000 | 100.6731 | 107.9269 |
|  |  | G4 | 38.90000^*^ | 1.04759 | .000 | 35.2731 | 42.5269 |
|  |  | G5 | 34.00000^*^ | 1.04759 | .000 | 30.3731 | 37.6269 |
|  |  | G6 | -21.70000^*^ | 1.04759 | .000 | -25.3269 | -18.0731 |
|  |  | G7 | 72.60000^*^ | 1.04759 | .000 | 68.9731 | 76.2269 |
| IL_10 | G1 | G2 | -144.00000^*^ | .93830 | .000 | -147.2485 | -140.7515 |
|  |  | G3 | -23.10000^*^ | .93830 | .000 | -26.3485 | -19.8515 |
|  |  | G4 | -74.40000^*^ | .93830 | .000 | -77.6485 | -71.1515 |
|  |  | G5 | -134.40000^*^ | .93830 | .000 | -137.6485 | -131.1515 |
|  |  | G6 | -180.80000^*^ | .93830 | .000 | -184.0485 | -177.5515 |
|  |  | G7 | -23.80000^*^ | .93830 | .000 | -27.0485 | -20.5515 |
|  |  | G8 | -163.40000^*^ | .93830 | .000 | -166.6485 | -160.1515 |
|  | G2 | G1 | 144.00000^*^ | .93830 | .000 | 140.7515 | 147.2485 |
|  |  | G3 | 120.90000^*^ | .93830 | .000 | 117.6515 | 124.1485 |
|  |  | G4 | 69.60000^*^ | .93830 | .000 | 66.3515 | 72.8485 |
|  |  | G5 | 9.60000^*^ | .93830 | .000 | 6.3515 | 12.8485 |
|  |  | G6 | -36.80000^*^ | .93830 | .000 | -40.0485 | -33.5515 |
|  |  | G7 | 120.20000^*^ | .93830 | .000 | 116.9515 | 123.4485 |
|  |  | G8 | -19.40000^*^ | .93830 | .000 | -22.6485 | -16.1515 |
|  | G3 | G1 | 23.10000^*^ | .93830 | .000 | 19.8515 | 26.3485 |
|  |  | G2 | -120.90000^*^ | .93830 | .000 | -124.1485 | -117.6515 |
|  |  | G4 | -51.30000^*^ | .93830 | .000 | -54.5485 | -48.0515 |
|  |  | G5 | -111.30000^*^ | .93830 | .000 | -114.5485 | -108.0515 |
|  |  | G6 | -157.70000^*^ | .93830 | .000 | -160.9485 | -154.4515 |
|  |  | G7 | -.70000 | .93830 | .994 | -3.9485 | 2.5485 |
|  |  | G8 | -140.30000^*^ | .93830 | .000 | -143.5485 | -137.0515 |
|  | G4 | G1 | 74.40000^*^ | .93830 | .000 | 71.1515 | 77.6485 |
|  |  | G2 | -69.60000^*^ | .93830 | .000 | -72.8485 | -66.3515 |
|  |  | G3 | 51.30000^*^ | .93830 | .000 | 48.0515 | 54.5485 |
|  |  | G5 | -60.00000^*^ | .93830 | .000 | -63.2485 | -56.7515 |
|  |  | G6 | -106.40000^*^ | .93830 | .000 | -109.6485 | -103.1515 |
|  |  | G7 | 50.60000^*^ | .93830 | .000 | 47.3515 | 53.8485 |
|  |  | G8 | -89.00000^*^ | .93830 | .000 | -92.2485 | -85.7515 |
|  | G5 | G1 | 134.40000^*^ | .93830 | .000 | 131.1515 | 137.6485 |
|  |  | G2 | -9.60000^*^ | .93830 | .000 | -12.8485 | -6.3515 |
|  |  | G3 | 111.30000^*^ | .93830 | .000 | 108.0515 | 114.5485 |
|  |  | G4 | 60.00000^*^ | .93830 | .000 | 56.7515 | 63.2485 |
|  |  | G6 | -46.40000^*^ | .93830 | .000 | -49.6485 | -43.1515 |
|  |  | G7 | 110.60000^*^ | .93830 | .000 | 107.3515 | 113.8485 |
|  |  | G8 | -29.00000^*^ | .93830 | .000 | -32.2485 | -25.7515 |
|  | G6 | G1 | 180.80000^*^ | .93830 | .000 | 177.5515 | 184.0485 |
|  |  | G2 | 36.80000^*^ | .93830 | .000 | 33.5515 | 40.0485 |
|  |  | G3 | 157.70000^*^ | .93830 | .000 | 154.4515 | 160.9485 |
|  |  | G4 | 106.40000^*^ | .93830 | .000 | 103.1515 | 109.6485 |
|  |  | G5 | 46.40000^*^ | .93830 | .000 | 43.1515 | 49.6485 |
|  |  | G7 | 157.00000^*^ | .93830 | .000 | 153.7515 | 160.2485 |
|  |  | G8 | 17.40000^*^ | .93830 | .000 | 14.1515 | 20.6485 |
|  | G7 | G1 | 23.80000^*^ | .93830 | .000 | 20.5515 | 27.0485 |
|  |  | G2 | -120.20000^*^ | .93830 | .000 | -123.4485 | -116.9515 |
|  |  | G3 | .70000 | .93830 | .994 | -2.5485 | 3.9485 |
|  |  | G4 | -50.60000^*^ | .93830 | .000 | -53.8485 | -47.3515 |
|  |  | G5 | -110.60000^*^ | .93830 | .000 | -113.8485 | -107.3515 |
|  |  | G6 | -157.00000^*^ | .93830 | .000 | -160.2485 | -153.7515 |
|  |  | G8 | -139.60000^*^ | .93830 | .000 | -142.8485 | -136.3515 |
|  | G8 | G1 | 163.40000^*^ | .93830 | .000 | 160.1515 | 166.6485 |
|  |  | G2 | 19.40000^*^ | .93830 | .000 | 16.1515 | 22.6485 |
|  |  | G3 | 140.30000^*^ | .93830 | .000 | 137.0515 | 143.5485 |
|  |  | G4 | 89.00000^*^ | .93830 | .000 | 85.7515 | 92.2485 |
|  |  | G5 | 29.00000^*^ | .93830 | .000 | 25.7515 | 32.2485 |
|  |  | G6 | -17.40000^*^ | .93830 | .000 | -20.6485 | -14.1515 |
|  |  | G7 | 139.60000^*^ | .93830 | .000 | 136.3515 | 142.8485 |
| *. The mean difference is significant at the 0.05 level. | | | | | | | |
